# Molecular sieving of iso-butene from C_4_ olefins with simultaneous high 1,3-butadiene and n-butene uptakes

**DOI:** 10.1038/s41467-024-46607-y

**Published:** 2024-03-12

**Authors:** Junhui Liu, Hanting Xiong, Hua Shuai, Xing Liu, Yong Peng, Lingmin Wang, Pengxiang Wang, Zhiwei Zhao, Zhenning Deng, Zhenyu Zhou, Jingwen Chen, Shixia Chen, Zheling Zeng, Shuguang Deng, Jun Wang

**Affiliations:** 1 Chemistry and Chemical Engineering School, Nanchang University, Nanchang, 330031 Jiangxi China; 2 School for Engineering of Matter, Transport and Energy, Arizona State University, Tempe, AZ 85287 USA

**Keywords:** Organic molecules in materials science, Organic-inorganic nanostructures, Organic molecules in materials science

## Abstract

Iso-butene (iso-C_4_H_8_) is an important raw material in chemical industry, whereas its efficient separation remains challenging due to similar molecular properties of C_4_ olefins. The ideal adsorbent should possess simultaneous high uptakes for 1,3-butadiene (C_4_H_6_) and n-butene (n-C_4_H_8_) counterparts, endowing high efficiency for iso-C_4_H_8_ separation in adsorption columns. Herein, a sulfate-pillared adsorbent, SOFOUR-DPDS-Ni (DPDS = 4,4′-dipyridyldisulfide), is reported for the efficient iso-C_4_H_8_ separation from binary and ternary C_4_ olefin mixtures. The rigidity in pore sizes and shapes of SOFOUR-DPDS-Ni exerts the molecular sieving of iso-C_4_H_8_, while exhibiting high C_4_H_6_ and n-C_4_H_8_ uptakes. The benchmark Henry’s selectivity for C_4_H_6_/iso-C_4_H_8_ (2321.8) and n-C_4_H_8_/iso-C_4_H_8_ (233.5) outperforms most reported adsorbents. Computational simulations reveal the strong interactions for C_4_H_6_ and n-C_4_H_8_. Furthermore, dynamic breakthrough experiments demonstrate the direct production of high-purity iso-C_4_H_8_ (>99.9%) from C_4_H_6_/iso-C_4_H_8_ (50/50, *v*/*v*), n-C_4_H_8_/iso-C_4_H_8_ (50/50, *v*/*v*), and C_4_H_6_/n-C_4_H_8_/iso-C_4_H_8_ (50/15/35, *v*/*v*/*v*) gas-mixtures.

## Abstract (web summary)

The efficient separation of iso-butene, an important raw material in chemical industry, remains challenging due to similar molecular properties of C_4_ olefins. Here, the authors report a sulfate-pillared adsorbent for the molecular sieving of iso-C_4_H_8_ from C_4_H_6_ and n-C_4_H_8_ with good benchmark uptake ratios and Henry’s selectivities.

## Introduction

Iso-butene (iso-C_4_H_8_) is a crucial feedstock for the production of butyl rubber, tert-butanol, and methyl tert-butyl ether (MTBE), with an annual consumption exceeding 30 million tons^1^. Generally, the steam cracking of naphtha generates C_4_ hydrocarbon mixtures containing 30~60% 1,3-butadiene (C_4_H_6_), 10~20% n-butene (n-C_4_H_8_), and 10~30% iso-C_4_H_8_^2^. While, the stringent purity requirement for polymer-grade iso-C_4_H_8_ (>99.5%) necessitates mandatory purification processes^3^. In industry, C_4_H_6_ is removed from C_4_ hydrocarbons in high extractive distillation towers (more than 110 trays) at harsh conditions of 3 bar and 323~393 K^4^. Furthermore, thermal-derived methods are inadequate for separating n-C_4_H_8_ and iso-C_4_H_8_ due to their subtle difference in boiling points (0.6 °C, Supplementary Table 2)^3^. The high-purity iso-C_4_H_8_ is obtained through the cracking of MTBE, the reaction product of iso-C_4_H_8_ fraction in C_4_ mixtures and methanol using strong acidic ion exchange resins as catalysts^2^. The excessive consumption of organic solvents and energy in traditional separation methods emphasizes the need for an energy- and cost-efficient strategy for iso-C_4_H_8_ purification from C_4_H_6_ and n-C_4_H_8_.

**Fig. 1:**
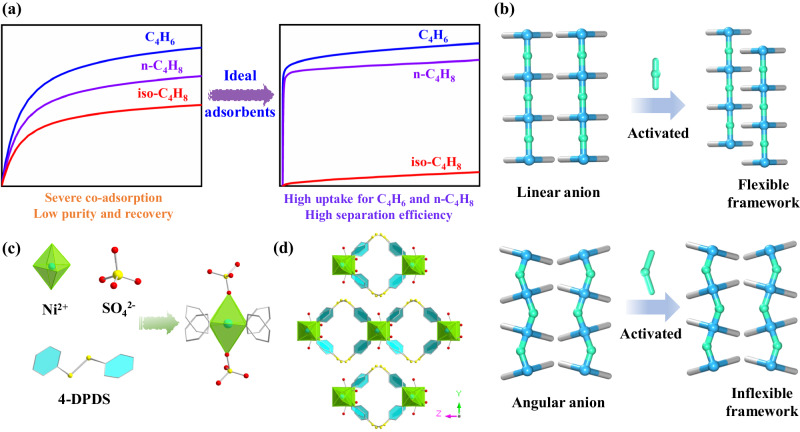
Scheme and structure of SOFOUR-DPDS-Ni. Schematic illustration of **a** adsorption behaviors on ideal adsorbents and **b** structure changes of adsorbents linked by linear and angular pillars after activation; **c** Building blocks (Ni^2+^, SO_4_^2−^, and DPDS organic ligand) and local coordination environment of metal atoms; **d** SOFOUR-DPDS-Ni structure along the *X-axes*.

Physisorption utilizing porous adsorbents, e.g., zeolites and metal-organic frameworks (MOFs)^5–13^, shows great promise in various challenging gas separations, including C_2_H_2_/C_2_H_4_, CO_2_/C_2_H_2_, and n-C_4_H_10_/iso-C_4_H_10_^14–22^. The development of advanced adsorbents capable of recognizing the subtle differences in molecular shapes, sizes, and properties among C_4_ counterparts remain a formidable challenge (Supplementary Fig. 1 and Supplementary Table 2). Namely, zeolite DD3R exhibited unsatisfactory adsorption capacities (0.832 mmol g^−1^ for C_4_H_6_), resulting in low separation efficiency^15^. In recent examples, adsorbents indiscriminately adsorbed C_4_ components with unsaturated bonds through open metal sites and high-polar pillars, causing significant co-adsorption and low iso-C_4_H_8_ recovery in fixed-bed columns^23,24^. The ideal adsorbent for iso-C_4_H_8_ purification from C_4_ olefins should possess high simultaneous adsorption capacities for both C_4_H_6_ and n-C_4_H_8_ while preventing the co-adsorption of iso-C_4_H_8_ in order to achieve optimal separation efficiency (Fig. 1a). To date, achieving complete molecular sieving of iso-C_4_H_8_ remains challenging for MOF adsorbents^1,24^. For example, Prof. Eddaoudi’s group reported Y-fum with a suitable aperture size of ~4.7 Å exhibited molecular sieving capabilities for iso-C_4_H_10_ and iso-C_5_H_12_ from their corresponding n-paraffins^25^. The successful synthesis of this type of adsorbent remains a formidable challenge, which is rarely reported thus far^26–28^.

Sulfate anions (SO_4_^2-^) possessing abundant lone pair electrons facilitate the formation of coordination bonds with metal ions^29^. Zaworotko et al., reported the first sulfate-pillared hybrid ultra-microporous adsorbent (SOFOUR-1-Zn) in 2021^30^. Our group has recently demonstrated the exceptional molecule recognition ability of SO_4_^2-^ groups in a sulfate-pillared adsorbent (SOFOUR-TEPE-Zn, TEPE = 1,1,2,2-tetra(pyridin-4-yl) ethene), which exhibited a benchmark selectivity of 16,833 for C_2_H_2_/CO_2_ separation^31^. For separation purposes, the rotation of linear pillars (e.g., SiF_6_^2−^, TiF_6_^2−^, GeF_6_^2−^) in anion-pillared adsorbents induced by interactions with adsorbates may compromise the yield and purity of iso-C_4_H_8_ due to its potential entrance and co-adsorption^20,24^. Moreover, the densely packed pore channels resulting from layer-to-layer sliding after solvent removal may lead to high gate-opening pressures, causing poor adsorption capacity and limited diffusion rate (Fig. 1b)^32,33^. In sharp contrast, the inflexibility of tetrahedral SO_4_^2-^ anions enables the preservation of pore shapes and sizes during activation and adsorption processes^34^, thereby conferring limited iso-C_4_H_8_ adsorption/diffusion in adsorbents.

Herein, we report a SO_4_^2−^-anion pillared adsorbent, SOFOUR-DPDS-Ni (DPDS = 4,4′-dipyridyldisulfide), for efficient iso-C_4_H_8_ separation from C_4_ olefins. The adsorption isotherms demonstrate simultaneous high uptakes of C_4_H_6_ and n-C_4_H_8_ while almost excluding iso-C_4_H_8_. Notably, the C_4_H_6_ adsorption capacity reaches a high value of 1.15 mmol g^−1^ at 0.001 bar (1000 ppm), indicating its potential for removing trace amounts of C_4_H_6_ from C_4_ olefin mixtures. The Henry’s selectivity is exceptional with values of 2321.8 and 233.5 for C_4_H_6_/iso-C_4_H_8_ and n-C_4_H_8_/iso-C_4_H_8_ at 298 K. Computational simulations confirm that C_4_H_6_ and n-C_4_H_8_ exhibit strong interactions with SO_4_^2−^ pillars and pyridyl rings through multiple C-H∙∙∙O, C-H∙∙∙C, and C-H∙∙∙H interactions. Charge bias analysis reveals the charge shifts of positive H atoms in C_4_H_6_ and n-C_4_H_8_ to negative states, while O atoms in SO_4_^2-^ pillars shift towards positive potential. Furthermore, dynamic breakthrough experiments demonstrate the direct production of high-purity iso-C_4_H_8_ (>99.9%) from binary and ternary components mixtures.

## Results

### Structure characterization

The reaction of NiSO_4_·6H_2_O and DPDS in methanol solutions at room temperature yielded light blue powder of SOFOUR-DPDS-Ni with the chemical formula of Ni(DPDS)_2_SO_4_ (Fig. 1c, see methods for details). Note that the synthesis must be conducted under anhydrous conditions as H_2_O molecules possess a significantly stronger coordination ability compared to SO_4_^2−^ anions, and may therefore occupy the coordination sites for Ni^2+^ ions^35^. Despite numerous attempts, high-quality single crystals of SOFOUR-DPDS-Ni could not be obtained for single crystal X-ray diffraction analysis. The Rietveld refinements of powder X-ray diffraction (PXRD) data revealed that the as-synthesized SOFOUR-DPDS-Ni crystallizes in the orthorhombic crystal system with specific cell parameters of a = 10.5377, b = 14.2957, c = 19.8289 (Supplementary Fig. 2 and Supplementary Table 8). The well matched PXRD and simulated XRD patterns confirmed the high phase-purity of bulk SOFOUR-DPDS-Ni (Supplementary Fig. 3). Each Ni^2+^ ion was coordinated with four pyridyl N atoms from four independent DPDS ligands in a distorted octahedral environment, forming one-dimensional chains of [Ni(DPDS)_2_]_n_ (Supplementary Fig. 4). These chains were further pillared by two SO_4_^2−^ anions in the axial direction, generating two-dimensional (2D) layers of [SOFOUR-DPDS-Ni]_n_ with the ***sql*** topology. Adjacent 2D layers are assembled into three-dimensional structures via multiple π-π stacking interactions among pyridyl rings and p-π interactions between S atoms and pyridyl rings (Fig. 1d)^36^. The pore sizes of resulting intralayer and interlayer channels were measured to be 3.3 × 4.5 and 3.6 × 3.7 Å^2^, respectively (Supplementary Fig. 5).

Thermogravimetric analysis (TGA) revealed the removal of guest molecules at 383 K, and demonstrated structural stability up to 473 K under N_2_ atmosphere (Supplementary Fig. 6). The permanent porosity of activated SOFOUR-DPDS-Ni was probed by CO_2_ gas sorption isotherms at 195 K, and the Brunauer-Emmett-Teller (BET) specific surface area was calculated to be 270 m^2^ g^−1^ with a total pore volume of 0.15 cm^3^ g^−1^ (Supplementary Fig. 7). Whereas, the N_2_ adsorption in SOFOUR-DPDS-Ni was limited at 77 K. Based on the Horvath-Kawazoe model, the experimental pore size distributions exhibited a centered pore size of approximately 4.7 Å (Supplementary Fig. 8), which was consistent with the dimensions of intralayer cavities (5.3 × 4.5 × 5.8 Å^3^) derived from the simulated crystal structure. The pore sizes of SOFOUR-DPDS-Ni were larger than the kinetic diameters of C_4_H_6_ (4.31 Å) and n-C_4_H_8_ (4.46 Å), yet smaller than that of iso-C_4_H_8_ (4.84 Å), suggesting the potential molecular sieving of iso-C_4_H_8_ from C_4_H_6_ and n-C_4_H_8_ counterparts. Additionally, the adsorption isotherms exhibited a high C_2_H_2_ uptake (2.87 mmol g^−1^) and the moderate CO_2_ uptake (1.36 mmol g^−1^) at 298 K and 1.0 bar (Supplementary Fig. 9). The C_2_H_2_/CO_2_ separation performance was considerably inferior compared to the molecular sieving effect on SO_4_^2−^-pillared SOFOUR-TEPE-Zn^31^.

The PXRD pattern remained unchanged after activation, indicating the rigidity of SOFOUR-DPDS-Ni (Supplementary Fig. 3). Due to the presence of angular SO_4_^2−^ pillars connecting adjacent 2D layers, the obtained unparallel stacking effectively prevented layer-to-layer sliding after removal of guest molecules (Fig. 1b). Additionally, no gate-opening or step-wise adsorption behavior was observed in the measured adsorption isotherms of C_4_H_6_ and n-C_4_H_8_ at different temperatures (Supplementary Fig. 10). The excellent chemical stability was demonstrated by its ability to withstand soaking in various organic solvents for one week, hot water at 60 °C for 2 h, and exposure to air for 13 months (Supplementary Fig. 11). The ultimate elemental analysis demonstrated that the element composition of each element corresponded well with the theoretical formula of SOFOUR-DPDS-Ni (C_20_H_16_N_4_O_4_S_5_Ni, Supplementary Table 1). For instance, the measured content ratio of N/S (0.39) was closely matched to the theoretical value (0.35). The scanning electron microscopy (SEM) image revealed a block morphology of SOFOUR-DPDS-Ni (Supplementary Fig. 12). Fourier transform infrared spectroscopy (FT-IR) spectra exhibited characteristic peaks corresponding to stretching vibrations for Ni-O at 493.2 cm^−1^ and S-O in SO_4_^2−^ at 1058.3 cm^−1^. Detailed discussions regarding FT-IR results are presented below Supplementary Fig. 13.

### Gas adsorption and separation behaviors

**Fig. 2:**
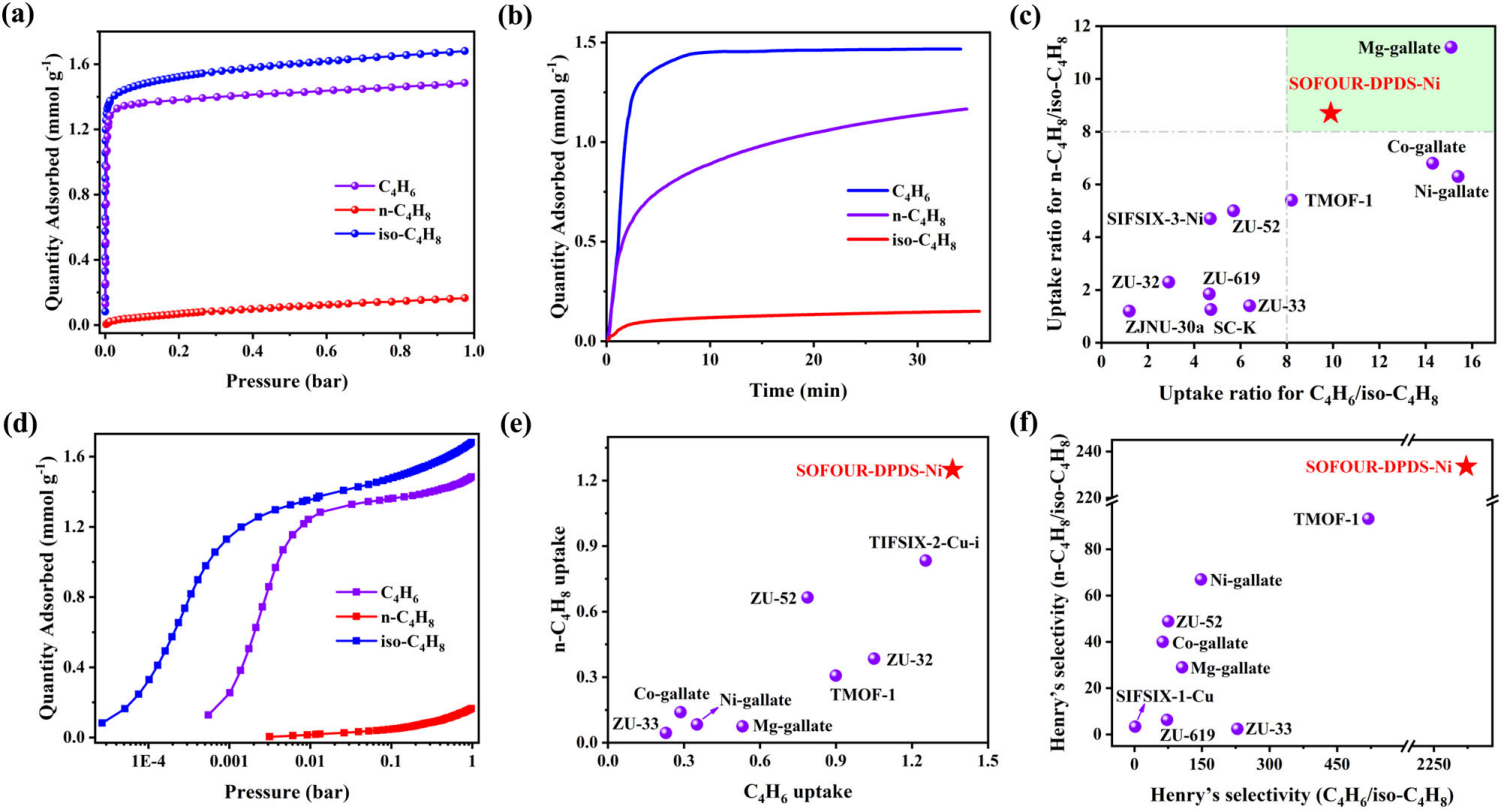
C_4_H_6_, n-C_4_H_8_, and iso-C_4_H_8_ sorption in SOFOUR-DPDS-Ni. **a** Pure-component isotherms at 298 K and **b** kinetic adsorption curves at 298 K and 0.5 bar for C_4_H_6_, n-C_4_H_8_, and iso-C_4_H_8_; **c** Comparison of uptake ratios of C_4_H_6_/iso-C_4_H_8_ and n-C_4_H_8_/iso-C_4_H_8_ at 1.0 bar; **d** Adsorption isotherms in logarithmic form for C_4_H_6_, n-C_4_H_8_, and iso-C_4_H_8_ at 298 K; **e** Comparison of uptakes of C_4_H_6_ and n-C_4_H_8_ at 0.01 bar; **f** Comparison of Henry’s selectivity for C_4_H_6_/iso-C_4_H_8_ and n-C_4_H_8_/iso-C_4_H_8_.

Single-component gas adsorption isotherms of C_4_H_6_, n-C_4_H_8_, and iso-C_4_H_8_ were collected on SOFOUR-DPDS-Ni (Fig. 2a and Supplementary Fig. 10). The adsorption capacity for C_4_H_6_ and n-C_4_H_8_ was measured to be 1.68 and 1.48 mmol g^−1^ at 298 K and 1.0 bar, respectively. In contrast, iso-C_4_H_8_ was almost completely excluded with a negligible uptake of only 0.17 mmol g^−1^. The kinetic adsorption curve disclosed that C_4_H_6_ reached adsorption equilibrium within 5 min, while n-C_4_H_8_ required a longer time of ~30 min to reach equilibrium (Fig. 2b). Whereas, iso-C_4_H_8_ exhibited negligible adsorption even after an extended exposure of 35 min. The kinetic adsorption capacity for C_4_H_6_ and n-C_4_H_8_ was determined to be 1.47 and 1.17 mmol g^−1^ at 0.5 bar, which were comparable to their static adsorption uptakes. The diffusion time constant (D/r^2^) was calculated to be 1.29 × 10^−3 ^s^−1^ for C_4_H_6_ and 2.72 × 10^−4 ^s^−1^ for n-C_4_H_8_, suggesting a faster diffusion rate for C_4_H_6_ in SOFOUR-DPDS-Ni (Supplementary Fig. 14). Furthermore, molecular dynamic (MD) simulations demonstrated that the diffusion coefficient of C_4_H_6_ (4.82 × 10^−11^) was faster than that of n-C_4_H_8_ (1.02 × 10^−11^) at 298 K (Supplementary Figs. 15 and 16). The kinetic separation selectivity of 4.7 for C_4_H_6_/n-C_4_H_8_ indicated a limited kinetic contribution to the apparent selectivity. Benefiting from the molecular sieving effect of iso-C_4_H_8_, uptake ratios were utilized as an intuitive measure of selectivity for separating C_4_ olefins. At 1.0 bar and 298 K, the uptake ratios of C_4_H_6_/iso-C_4_H_8_ and n-C_4_H_8_/iso-C_4_H_8_ on SOFOUR-DPDS-Ni were calculated to be 9.9 and 8.7, surpassing most top-ranking adsorbents including TMOF-1 (8.2 and 5.4)^1^, ZU-52 (5.7 and 5.0)^24^, and SIFSIX-3-Ni (4.7 and 4.7)^24^ (Fig. 2c and Supplementary Table 6). In comparison, we measured the adsorption isotherms of C_4_ olefins on SOFOUR-1-Zn and SOFOUR-TEPE-Zn at 298 K (Supplementary Fig. 17). SOFOUR-1-Zn adsorbed comparable uptakes for n-C_4_H_8_ (0.72 mmol g^−1^) and iso-C_4_H_8_ (0.55 mmol g^−1^). Meanwhile, negligible n-C_4_H_8_ and iso-C_4_H_8_ uptakes (<0.13 mmol g^−1^) were observed on SOFOUR-TEPE-Zn. These inferior performances on SO_4_^2−^-pillared adsorbents further highlighted the advantages of pore environments of SOFOUR-DPDS-Ni.

Remarkably, SOFOUR-DPDS-Ni exhibited high adsorption capacity of 1.36 mmol g^−1^ for C_4_H_6_ and 1.25 mmol g^−1^ for n-C_4_H_8_ at a low pressure of 0.01 bar (Fig. 2d). At even lower pressure of 0.001 bar (1000 ppm), the C_4_H_6_ uptake unprecedently reached 1.15 mmol g^−1^, suggesting its potential removal of trace C_4_H_6_ from C_4_ olefin mixtures. The adsorption affinities for C_4_H_6_ and n-C_4_H_8_ were further confirmed by the isosteric heats of adsorption (*Q*_*st*_) using the Clausius-Clapeyron equation^37^ (Supplementary Fig. 18). Specifically, the *Q*_*st*_ was calculated to be 77 kJ mol^−1^ and 38 kJ mol^−1^ for C_4_H_6_ and n-C_4_H_8_ at near zero coverage, respectively (Supplementary Fig. 19). The high uptakes of both C_4_H_6_ and n-C_4_H_8_ established a new benchmark for iso-C_4_H_8_ separation, outperforming most adsorbents including ZU-32^24^, TMOF-1 ^1^, and Ni-gallate^38^ (Fig. 2e). Moreover, the Henry’s constant of C_4_H_6_ and n-C_4_H_8_ was calculated to be 50.87 and 5.11 mmol g^−1^ kPa^−1^, which were significantly higher than that of iso-C_4_H_8_ (0.022 mmol g^−1^ kPa^−1^). As a result, the Henry’s selectivity for C_4_H_6_/iso-C_4_H_8_ and n-C_4_H_8_/iso-C_4_H_8_ on SOFOUR-DPDS-Ni reached high values of 2321.8 and 233.5 at 298 K, respectively. As shown in Fig. 2f, these values were much higher than other reported adsorbents such as TMOF-1 (519.2 and 93.2)^1^, ZU-33 (228.7 and 2.4)^24^, and ZU-52 (75.2 and 48.9)^24^ (Supplementary Table 7).

### Simulation studies

**Fig. 3:**
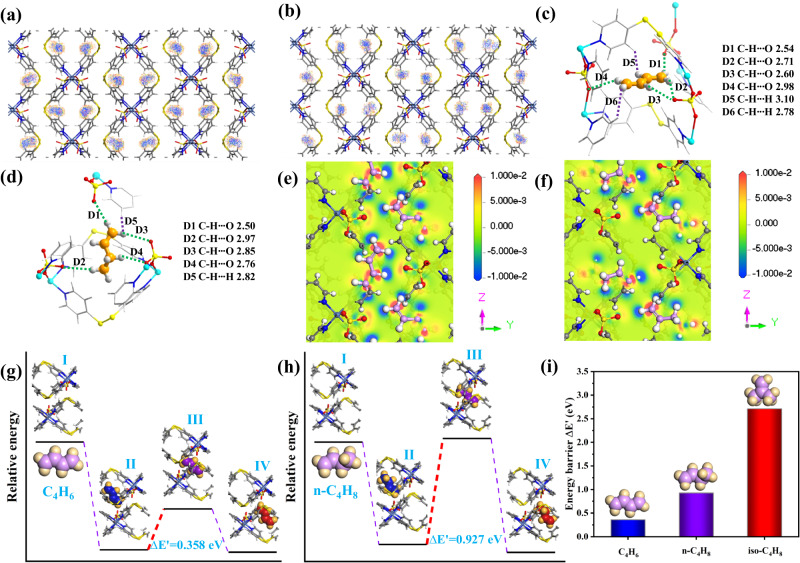
GCMC simulated and DFT-D calculated results in SOFOUR-DPDS-Ni. Distribution density for **a** C_4_H_6_ and **b** n-C_4_H_8_ in SOFOUR-DPDS-Ni at 1.0 bar; Adsorption binding site for **c** C_4_H_6_ and **d** n-C_4_H_8_ in SOFOUR-DPDS-Ni; Charge density difference plots of **e** C_4_H_6_-loaded and **f** n-C_4_H_8_-loaded structure; Illustration of diffusion pathway and corresponding energy levels **g** C_4_H_6_ and **h** n-C_4_H_8_; **i** Comparison of diffusion energy barriers for C_4_H_6_, n-C_4_H_8_, and iso-C_4_H_8_.

Grand Canonical Monte Carlo (GCMC) and first-principles dispersion-corrected density function theory (DFT-D) simulations were employed to elucidate the adsorption mechanism of C_4_ olefins on SOFOUR-DPDS-Ni. The distribution densities of C_4_H_6_ and n-C_4_H_8_ were investigated via GCMC simulations at 0.01 bar and 1.0 bar, both gas molecules were adsorbed in the intralayer spaces near SO_4_^2-^ pillars (Fig. 3a, b). It is noteworthy that the distribution densities of C_4_H_6_ and n-C_4_H_8_ were nearly identical at pressures of 0.01 and 1.0 bar (Supplementary Figs. 21 and 22), which aligned with their early adsorption saturation at low pressures in SOFOUR-DPDS-Ni. Moreover, four C_4_H_6_ or n-C_4_H_8_ molecules were adsorbed per unit cell, their uptakes were accordingly calculated to be 1.68 mmol g^−1^ at 1.0 bar, which were closely matched to their experimental uptakes.

DFT-D simulations revealed two favorable adsorption sites for C_4_H_6_ in the interlayer cavities of SOFOUR-DPDS-Ni, which were captured by SO_4_^2−^ anions through multiple C-H∙∙∙O interactions with distances of 2.41–2.98 Å and pyridine rings through multiple van der Walls interactions of C-H∙∙∙C and C-H∙∙∙H with distances of 2.77–3.10 Å (Fig. 3c and Supplementary Fig. 23a). The calculated static binding energy (∆E) for Site I and Site II were estimated to be 78.3 and 80.6 kJ mol^−1^, respectively (Supplementary Fig. 24). Due to the conformational changes of n-C_4_H_8_, four favorable adsorption sites for n-C_4_H_8_ were identified, while the resulting interactions were comparable to those of C_4_H_6_. Specifically, n-C_4_H_8_ molecules strongly interacted with SO_4_^2−^ anions through multiple C-H∙∙∙O interactions with distances of 2.43–2.98 Å and pyridine rings through multiple van der Walls interactions of C-H∙∙∙C and C-H∙∙∙H with distances of 2.24–2.95 Å (Fig. 3d and Supplementary Fig. 23b–d). The calculated ∆E values for the four binding sites were 72.8, 58.3, 64.6, and 71.2 kJ mol^−1^, respectively (Supplementary Fig. 24). Furthermore, gas-loaded structures were subjected to charge transfer analysis, with the blue and yellow surfaces indicating charge accumulation and depletion, respectively. Upon adsorption, the initial positively charged H atoms in C_4_H_6_ and n-C_4_H_8_ were shifted to strong negative potentials, while O atoms in SO_4_^2-^ pillars shifted to positive potential (Fig. 3e, f). In addition, charge transfer also occurred between the adsorbed guests and the pyridine rings (Supplementary Fig. 25). Therefore, the strong interaction forces between [SOFOUR-DPDS-Ni]_n_ layers (i.e., SO_4_^2−^ pillars and pyridyl rings) and adsorbed guests (i.e., C_4_H_6_ and n-C_4_H_8_) stabilized the framework of SOFOUR-DPDS-Ni, thereby endowing it with rigidity during C_4_ adsorptions.

The energy levels associated with the diffusions processes of C_4_H_6_, n-C_4_H_8_, and iso-C_4_H_8_ into SOFOUR-DPDS-Ni framework were further investigated. The rate-determining step was attributed to the largest energy input required for the energy barrier of transition-states (TS) between surface adsorption TS (II) and diffusion into pores TS (III). The diffusion energy barrier of C_4_H_6_ in SOFOUR-DPDS-Ni was determined to be 0.358 eV, indicating its facile transport through interlayer channels (Fig. 3g). The diffusion energy barrier increased to 0.927 eV for n-C_4_H_8_, attributing to its larger steric hindrance caused by the methyl group (Fig. 3h). In contrast, the overwhelming diffusion energy barrier for iso-C_4_H_8_ (2.712 eV) suggested inhibited diffusions thus causing the negligible adsorption amount (Fig. 3i and Supplementary Fig. 26).

### Transient breakthrough experiments

**Fig. 4:**
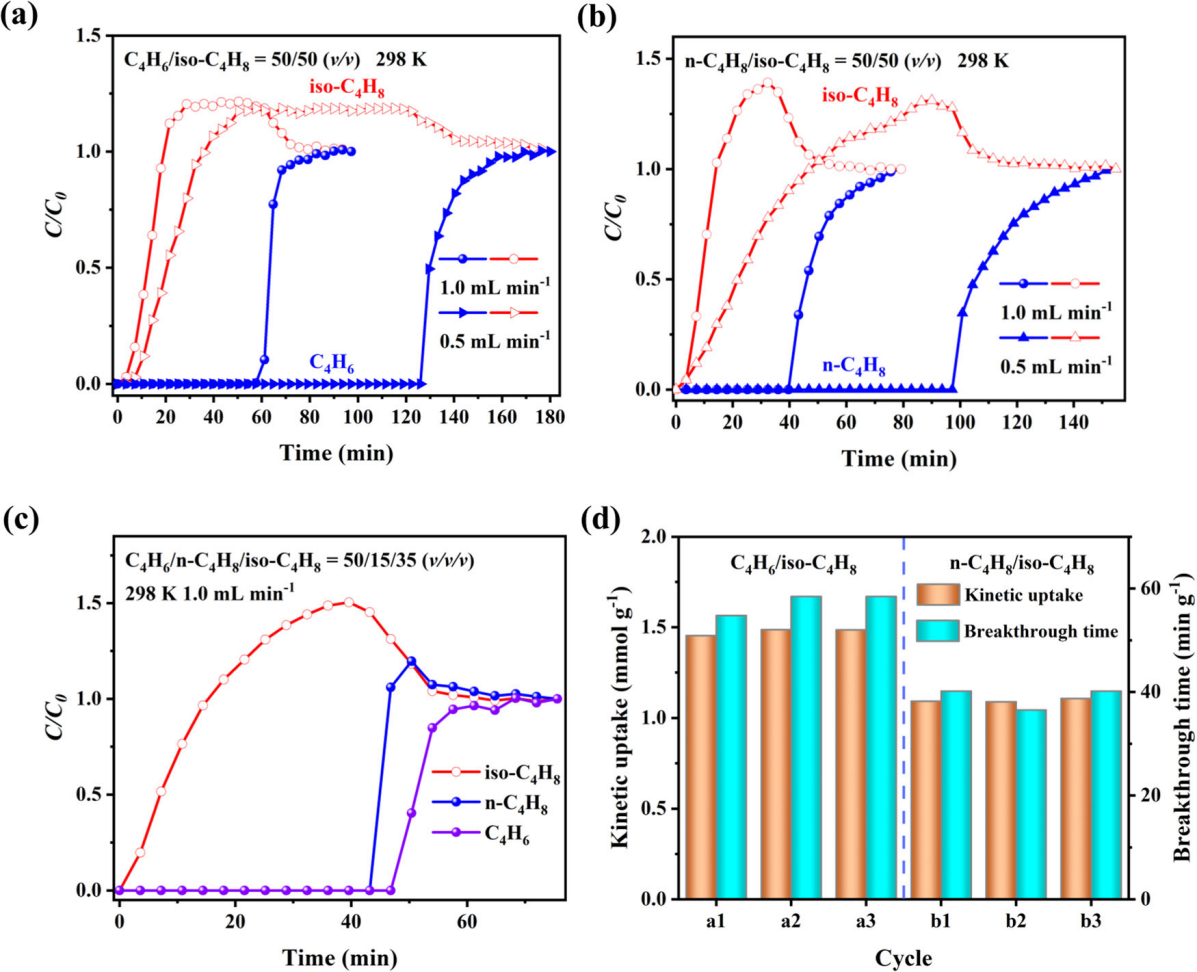
C_4_H_6_, n-C_4_H_8_, and iso-C_4_H_8_ separation performances. Experimental column breakthrough curves for **a** C_4_H_6_/iso-C_4_H_8_ (50/50, *v*/*v*), **b** n-C_4_H_8_/iso-C_4_H_8_ (50/50, *v*/*v*), and **c** C_4_H_6_/n-C_4_H_8_/iso-C_4_H_8_ (50/15/35, *v*/*v*/*v*); **d** Dynamic uptakes for three cycling breakthrough tests for C_4_H_6_/iso-C_4_H_8_ (a1-a3) and n-C_4_H_8_/iso-C_4_H_8_ (b1-b3) at 1.0 mL min^−1^.

Transient breakthrough experiments were conducted to evaluate the practical separation performances of SOFOUR-DPDS-Ni for C_4_ mixtures^39^, which exhibit varying component proportions due to diverse streams from different stream cracking processes (Supplementary Fig. 27). Here, C_4_ olefin gas-mixtures of C_4_H_6_/iso-C_4_H_8_ (50/50, *v/v*), n-C_4_H_8_/iso-C_4_H_8_ (50/50, *v/v*), and C_4_H_6_/n-C_4_H_8_/iso-C_4_H_8_ (50/15/35, *v/v/v*) were selected as representative gas-mixtures for simulating the actual separation process. For C_4_H_6_/iso-C_4_H_8_ mixture with a flow rate of 1.0 mL min^−1^, iso-C_4_H_8_ was eluted first at the outlet of packed column, while C_4_H_6_ was not detected until 57.6 min (Fig. 4a). The kinetic adsorption capacity of C_4_H_6_ on SOFOUR-DPDS-Ni was calculated to be 1.48 mmol g^−1^, which closely approximated its static uptake of 1.59 mmol g^−1^ at 0.5 bar. High-purity iso-C_4_H_8_ (>99.9%) could be collected at the outlet with a productivity of 1.20 mmol g^−1^. Furthermore, high-purity C_4_H_6_ (>99.0%) could be collected from 14.4 min during the desorption process with a productivity of 1.17 mmol g^−1^ using He purge at 5.0 mL min^−1^ and 343 K (Supplementary Fig. 28). The efficient separation of C_4_H_6_/iso-C_4_H_8_ could also be achieved at a slower flow rate of 0.5 mL min^−1^ with a comparable kinetic C_4_H_6_ uptake of 1.51 mmol g^−1^. Similarly, for n-C_4_H_8_/iso-C_4_H_8_ gas-mixture, iso-C_4_H_8_ immediately broke through the column, followed by n-C_4_H_8_ at 39.6 min at 1.0 mL min^−1^ (Fig. 4b). The dynamic uptake of n-C_4_H_8_ was determined to be 1.09 mmol g^−1^, which was 76.8% of its static adsorption uptake (1.42 mmol g^−1^) at 0.5 bar. High-purity iso-C_4_H_8_ (>99.9%) effluent could be obtained with a productivity of 0.85 mmol g^−1^. While high-purity n-C_4_H_8_ (>99.0%) could also be collected during the desorption process with a productivity of 0.74 mmol g^−1^ (Supplementary Fig. 29). The kinetic uptake of n-C_4_H_8_ increased to 1.28 mmol g^−1^ (90.1% of static uptake) at 0.5 mL min^−1^, indicating the slower diffusion rate of n-C_4_H_8_ compared to that of C_4_H_6_.

Moreover, efficient separation of a ternary gas-mixture containing C_4_H_6_/n-C_4_H_8_/iso-C_4_H_8_ (50/15/35, *v/v/v*) at 1.0 mL min^−1^ was successfully achieved using SOFOUR-DPDS-Ni as well. Figure 4c depicted that iso-C_4_H_8_ was immediately detected, while C_4_H_6_ and n-C_4_H_8_ were retained in the column for 46.8 min and 43.2 min, respectively. Their competitive adsorptions will simultaneously occupy the available adsorption sites and inevitably influence their adsorption uptakes. High-purity iso-C_4_H_8_ (>99.9%) could be directly collected with a productivity of 0.71 mmol g^−1^ during a time period of 43.2 min. The reusability of SOFOUR-DPDS-Ni was validated through three successive breakthrough cycles using binary and ternary C_4_ gas-mixtures (Supplementary Figs. 30–35). The cycling breakthrough points and curve shapes were almost overlapped, indicating consistent separation performance. Furthermore, the kinetic adsorption capacity for C_4_H_6_ and n-C_4_H_8_ also remained consistent at 0.5 and 1.0 mL min^−1^ throughout the cycling process (Fig. 4d and Supplementary Fig. 36). Furthermore, the PXRD patterns of SOFOUR-DPDS-Ni remained change after cycled adsorption tests and breakthrough experiments, thereby indicating its exceptional structural stability (Supplementary Fig. 39).

## Discussion

In summary, we successfully synthesized the sulfate-pillared adsorbent, SOFOUR-DPDS-Ni, for efficient separation of iso-C_4_H_8_ from C_4_ olefin mixtures. Simultaneous high uptakes for C_4_H_6_ and n-C_4_H_8_ of 1.68 and 1.48 mmol g^−1^ at 298 K and 1.0 bar were achieved on SOFOUR-DPDS-Ni, while exhibiting size-sieving effect for iso-C_4_H_8_. At low pressures of 0.01 bar, SOFOUR-DPDS-Ni exhibited high adsorption capacity of 1.36 mmol g^−1^ for C_4_H_6_ and 1.25 mmol g^−1^ for n-C_4_H_8_. Notably, at an ultralow pressure of 0.001 bar (1000 ppm), the unprecedented C_4_H_6_ adsorption capacity of 1.15 mmol g^−1^ indicated the potential for trace C_4_H_6_ removal. Therefore, the benchmark uptake ratio and Henry’s selectivity for C_4_H_6_/iso-C_4_H_8_ (9.9 and 2321.8) and n-C_4_H_8_/iso-C_4_H_8_ (8.7 and 233.5) surpassed most top-ranking adsorbents. GCMC and DFT-D simulations demonstrated the adsorption sites and separation mechanisms. Breakthrough experiments confirmed the practical iso-C_4_H_8_ separation performances of SOFOUR-DPDS-Ni from binary and ternary C_4_ olefins mixtures. The high-purity iso-C_4_H_8_ (99.9%) could be directly collected, meanwhile high-purity C_4_H_6_ (99.0%) and n-C_4_H_8_ (99.0%) could also be obtained during desorption processes.

## Methods

### Materials

All reagents and solvents were obtained from commercial sources and used without further purification. Nickel sulfate hexahydrate (NiSO_4_·6H_2_O, 99.0%, Aladdin), 4,4’-dipyridyl disulfide (C_10_H_8_N_2_S_2_, 98.0%, Xiya Reagent), and methanol (CH_4_O, anhydrous, 99.9%, Aladdin). 1,3-butadiene (C_4_H_6_, 99.9%), n-butene (n-C_4_H_8_, 99.9%), iso-butene (iso-C_4_H_8_, 99.9%), N_2_ (99.999%), He (99.999%), and mixed gas-mixtures of C_4_H_6_/iso-C_4_H_8_ (50/50, *v/v*), n-C_4_H_8_/iso-C_4_H_8_ (50/50, *v/v*), and C_4_H_6_/n-C_4_H_8_/iso-C_4_H_8_ (50/15/35, *v/v/v*) were purchased from Nanchang Jiangzhu Gas Co., Ltd (China).

### Synthesis of SOFOUR-DPDS-Ni

The material was designated as SOFOUR-DPDS-Ni, with a chemical formula of Ni(DPDS)_2_SO_4_. NiSO_4_·6H_2_O (0.2 mmol, 0.0526 g) was added to a solution of 4-DPDS (0.4 mmol, 0.0881 g) in 20 mL MeOH and stirred at room temperature for 24 h. SOFOUR-DPDS-Ni was obtained as a light blue powder and washed with 100 mL MeOH, followed by drying for 6 h at room temperature.

### Details for Rietveld refinement

We applied the EXPO2014 software to conduct the Rietveld refinement, the 2θ range of 5~60° was used for the refinement. Chebyshev (Background Function) and Pseudo-Voigt (Peak Shape Functions) were applied to refine the structure until the R_wp_ value converged and the overlay of the observed with refined profiles showed good agreement. Unit cell parameters and fitting reliability are listed in Supplementary Table 8, and we have deposited the CIF in the CCDC database with an identifier number of 2260840.

### Characterizations

Powder X-ray diffraction (PXRD) analysis of powder samples was carried out on a PANalytical Empyrean Series 2 diffractometer with Cu Kα radiation ((λ = 1.540598 Å), which operated at 40 kV, 40 mA and a scan speed of 0.0167°, a scan time of 15 s per step and 2θ ranging from 5 to 60° at room temperature. The thermogravimetric analysis (TGA) data were obtained on a NETZSCH Thermogravimetric Analyzer (STA2500) from 25 to 800 °C with a heating rate of 20 °C min^−1^ under an N_2_ atmosphere. The contents of C, H, N, and S elements were determined by an Elementar Vario MICRO elemental analyzer with CHNS measurement mode. The SEM images were recorded on a Thermo Scientific Apreo 2C scanning electron microscope with an accelerated voltage of 10 kV. The Fourier transfer infrared spectroscopy (FT-IR) was tested by NICOLET FT-IR spectrometer (iS50 FI-IR), and the resolution was 4 cm^−1^, the number of scans was 32, and the test wave number was 400–4000 cm^−1^.

### Gas adsorption measurements

Single-component isotherms of C_4_H_6_, n-C_4_H_8_, and iso-C_4_H_8_ were measured up to 1 bar at 283 and 298 K on Micromeritics 3Flex adsorption apparatus (Micromeritics Instruments, USA). The kinetic adsorptions of C_4_H_6_, n-C_4_H_8_, and iso-C_4_H_8_ were obtained on Intelligent Gravimetric Analyzer (IGA−100, HIDEN), and the pressure rise rate is 200 mbar min^−1^. About 100 mg powder samples were evacuated under high vacuum (<5 μm of Hg) at 70 °C for 12 h before adsorption measurement, and the free space of the system was measured by using helium gas. Liquid nitrogen and dry ice were used for adsorption isotherms at 77 K and 195 K, the pore size distribution was calculated based on CO_2_ adsorption isotherms at 195 K.

### Transient breakthrough experiments

The breakthrough experiments were implemented in a stainless-steel column (4.6 mm inner diameter ×200 mm) manually packed with the weight of 0.9853 g activated SOFOUR-DPDS-Ni. The column was first purged with a He flow (10 mL min^−1^) at room temperature for 10 h before breakthrough tests. The mixtures of C_4_H_6_/iso-C_4_H_8_ (50/50, *v/v*), n-C_4_H_8_/iso-C_4_H_8_ (50/50, *v/v*), and C_4_H_6_/n-C_4_H_8_/iso-C_4_H_8_ (50/15/35, *v/v/v*) was conducted at a flow rate of 0.5 and 1.0 mL min^−1^, respectively. The outlet gas from the column was monitored using gas chromatography (Panna A91 Plus GC) for continuous sampling gas analysis, and an attached mass flow controller (Seven Star, MC-2SCCM-D, D07 series) was used to control the gas flow rate. After the breakthrough tests, the columns packed with samples were regenerated by purging He gas of 10 mL min^−1^ at 70 °C for 8 h. For the C_4_ mixtures of C_4_H_6_/iso-C_4_H_8_ (50/50, *v/v*) and n-C_4_H_8_/iso-C_4_H_8_ (50/50, *v/v*), desorption process was carried out in order to obtain high-purity C_4_H_6_ and n-C_4_H_8_ after the breakthrough experiment. The desorption process was conducted under He gas flow rate of 5.0 mL min^−1^ at 70 °C.

### Structural stability tests

The activated samples of about 100 mg were placed in 20 mL vials containing 10 mL different solvents for 7 days, treated in hot water of 60 °C for 2 h, exposed air for thirteen months, respectively. The treated samples were washed with 100 mL MeOH and dried at room temperatures, and then characterized by PXRD measurements to determine whether the sample retains structural integrity.

### Grand Canonical Monte Carlo (GCMC) calculations

All the GCMC simulations were performed in Materials Studio package. The framework, C_4_H_6_, and n-C_4_H_8_ were considered to be rigid during the simulation. The charges for atoms of the SOFOUR-DPDS-Ni and gas components were derived from the Mulliken method. The simulations adopted the fixed pressure task, Metropolis method in sorption module, and the universal force field (UFF). The interaction energy between the adsorbed molecules and the framework was computed through Lennard-Jones 6−12 (LJ) potentials. The cutoff radius was chosen 15.5 Å and the electrostatic interactions were handled using the Ewald summation method. The loading steps and the equilibration steps were 1 × 10^7^, the production steps were 1 × 10^7^.

### Density Functional Theory calculations

First-principles density functional theory (DFT) calculations were performed using the Materials Studio’s CASTEP code. All calculations were conducted under the generalized gradient approximation (GGA) with Perdew–Burke–Ernzerhof (PBE). A semiempirical addition of dispersive forces to conventional DFT was included in the calculation to account for van der Waals interactions. The total energy coverage within 0.01 meV atom^−1^. The optimization process commenced with refine structures of the synthesized materials. The charge transfer analysis on gas-loaded structures was calculated using “Electron density difference” in properties of CASTEP module. Single point energy calculations using Dmol^3^ module. To obtain the binding energy, an isolated gas molecule placed in a cell unit (with the same cell dimensions as the MOF crystal). The static binding energy was calculated by the equation:

1 $$\Delta E=E\left({{{{{\rm{gas}}}}}}\right)+E\left({{{{{\rm{adsorbent}}}}}}\right)-E\left({{{{{\rm{adsorbent}}}}}}+{{{{{\rm{gas}}}}}}\right)$$

### The energy barrier calculation method

The energy barrier calculations were carried out using the Dmol^3^ module in Materials Studio. The unit cells were optimized until the force acting between atoms was below 0.002 Ha Å^−1^ with SCF convergence of 10^−6^. The Global orbital cutoff was 5.2 Å. The diffusion of guest molecules was studied by determining the transition state energies using the climbing nudged elastic band (cNEB) method. Firstly, the surface model and the host structure would be optimized using the refine structures as initial geometries with full structural relaxation. The isolated guest molecules (C_4_H_6_, n-C_4_H_8_, and iso-C_4_H_8_) were placed in unit cell and relaxed as references. Next, the guest molecules were introduced onto the host surface and different locations in the channel pore of the host structure, respectively, followed by a full structural relaxation. Then the optimized configurations of the lowest energy were utilized for the subsequent analysis and calculation. The transition state search calculations were used to capture the transition states with guest transport between the two energy minimum configurations from the host surface to channels.

The energy barrier was determined using the following:

2 $$\Delta {E}^{{\prime} }=E\left({{{{{\rm{Transition\; State}}}}}}\right)-E\left({{{{{\rm{Initial\; State}}}}}}\right)$$

where *E*(Transition State) is the transition energy, *E*(Initial State) is the energy of the optimized host-guest structure where guests were introduced onto the host surface.

### Calculation of selectivity

The single-component adsorption isotherms of C_4_ hydrocarbons were correlated by the Langmuir model at low pressure of 0–5 kPa. The Langmuir model was defined as:

3 $$q=\frac{{q}_{m}{bp}}{1+{bp}}$$

where, *q* is the adsorbed amount of the pure component i (mmol g^−1^), *p* is the pressure of the bulk gas at equilibrium (kPa), *q*_*m*_ is the saturated adsorption capacities (mmol g^−1^), *b* is the affinity parameters of the pure component (kPa^−1^).

To estimate the separation selectivity of SOFOUR-DPDS-Ni, Henry’s selectivity (*α*_*ij*_) was developed and applied, which reflected separation selectivity at low pressure about 0–5 kPa. The Henry’s selectivity (*α*_*ij*_) based on equilibrium alone can be calculate from the ratio of Henry’s constants, *H=q*_*m*_*×b*. The selectivity was defined by the following equation:

4 $${\alpha }_{{ij}}=\frac{{H}_{i}}{{H}_{j}}=\frac{{q}_{{mi}}{b}_{i}}{{q}_{{mj}}{b}_{j}}$$

### Isosteric heat of adsorption

The experiment isosteric heat of adsorption for C_4_H_6_ and n-C_4_H_8_ were calculated using the data at 283 K and 298 K, which was calculated by the Clausius–Clapeyron equation and was defined as:

5 $${{{\mbox{Q}}}}_{{{\mbox{st}}}}=-{{\mbox{R}}}{{{\mbox{T}}}}^{2}\left(\frac{\partial {{{{\mathrm{ln}}}}}{{\mbox{P}}}}{\partial {{\mbox{T}}}}\right)$$

where *Q*_*st*_ (kJ mol^−1^) represents the adsorption heat of gas molecular, *P* (mmHg) and *T* (K) represent the pressure and temperature, respectively, and *R* is the universal gas constant. Here, the adsorption heat of each component was determined precisely according to the virial fitting parameters of single-component adsorption isotherms measured at 283 and 298 K, which was calculated as follows:

6 $${InP}={InN}+\frac{1}{T}\mathop{\sum }\limits_{i=0}^{m}{a}_{i}{N}^{i}+\mathop{\sum }\limits_{j=0}^{n}{b}_{j}{N}^{j}$$

7 $${Q}_{{st}}=-R\mathop{\sum }\limits_{i=0}^{m}{a}_{i}{N}^{i}$$

where the *N* (mg g^−1^) is the adsorption amount, and *m* and *n* determine the number of items required to precisely fit the adsorption isotherms.

### Calculation of kinetic adsorption

The diffusional time constants (*D’*, *D/r*^2^) were calculated by the short-time solution of the diffusion equation assuming a step change in the gas-phase concentration, clean beds initially and micropore diffusion control:

8 $$\frac{{M}_{t}}{{M}_{e}}=\frac{6}{\sqrt{\pi }}\cdot \sqrt{\frac{D}{{r}^{2}}\cdot t}$$

where *t* (s) is the time, *M*_*t*_ (mmol g^−1^) is gas uptake at time *t*, *M*_*e*_ (mmol g^−1^) is the gas uptake at equilibrium, *D* (m^2^ s^−1^) is the diffusivity and *r* (m) is the radius of the equivalent spherical particle. The slopes of *M*_*t*_*/M*_*e*_ versus *t*^*1/2*^ are derived from the fitting of the plots at 0.5 bar and 298 K, and the pressure rise rate is 200 mbar min^−1^.

### Supplementary information

Supplementary Information

Peer Review File

## Data Availability

All data supporting the finding of this study are available within this article and its Supplementary Information. Crystallographic data for the structure in this article have been deposited at the Cambridge Crystallographic Date Center under deposition nos. CCDC 2260840 (SOFOUR-DPDS-Ni). Correspondence and requests for materials should be addressed to J.W.
