# Supplementary material for: Molecular sieving of iso-butene from C4 olefins with simultaneous high 1,3-butadiene and n-butene uptakes
Source: Nat Commun. 2024 Mar 12;15:2222. doi: 10.1038/s41467-024-46607-y (PMC10933363; doi:10.1038/s41467-024-46607-y)
Supplement: Supplementary file 1 — Supplementary Information [file 41467_2024_46607_MOESM1_ESM.pdf]

Supplementary Information

Molecular sieving of iso-butene from C_4 olefins with simultaneous high 1,3-butadiene and n-butene uptakes

Junhui Liu[1], Hanting Xiong[1], Hua Shuai[1], Xing Liu[1], Yong Peng[1], Lingmin Wang[1], Pengxiang Wang[1], Zhiwei Zhao[1], Zhenning Deng[1], Zhenyu Zhou[1], Jingwen Chen[1], Shixia Chen[1], Zheling Zeng[1], Shuguang Deng[2], Jun Wang[1]*

1. Chemistry and Chemical Engineering School, Nanchang University, Nanchang, Jiangxi 330031, China
2. School for Engineering of Matter, Transport and Energy, Arizona State University, Tempe, Arizona 85287, United States

*Corresponding author.

E-mail addresses: jwang7@ncu.edu.cn (J. Wang)

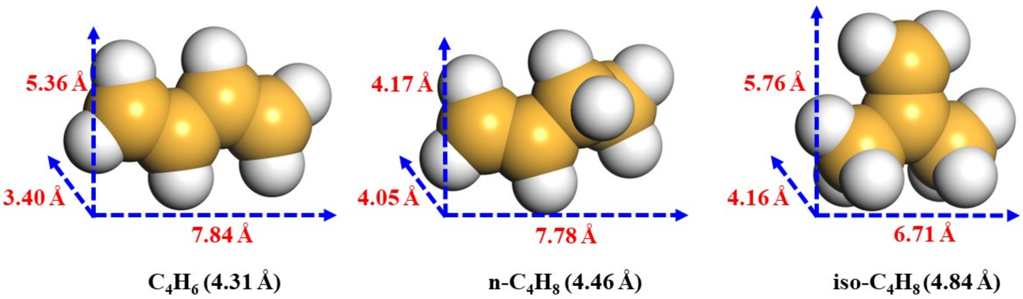

Supplementary Figure 1. Molecular size. Comparison of the sizes of C_4H_6, n-C_4H_8, and iso-C_4H_8.

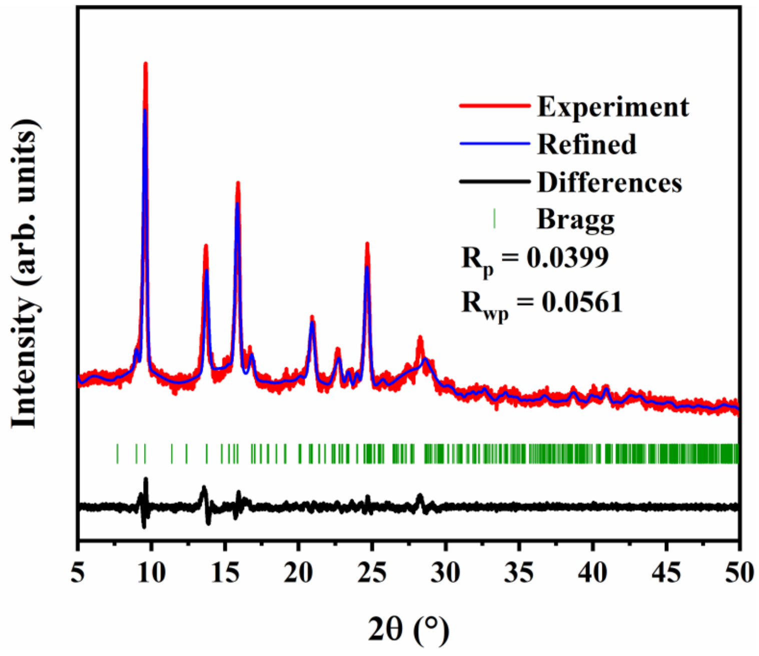

Supplementary Figure 2. Rietveld refinement. PXRD Rietveld refinement for SOFOUR-DPDS-Ni.

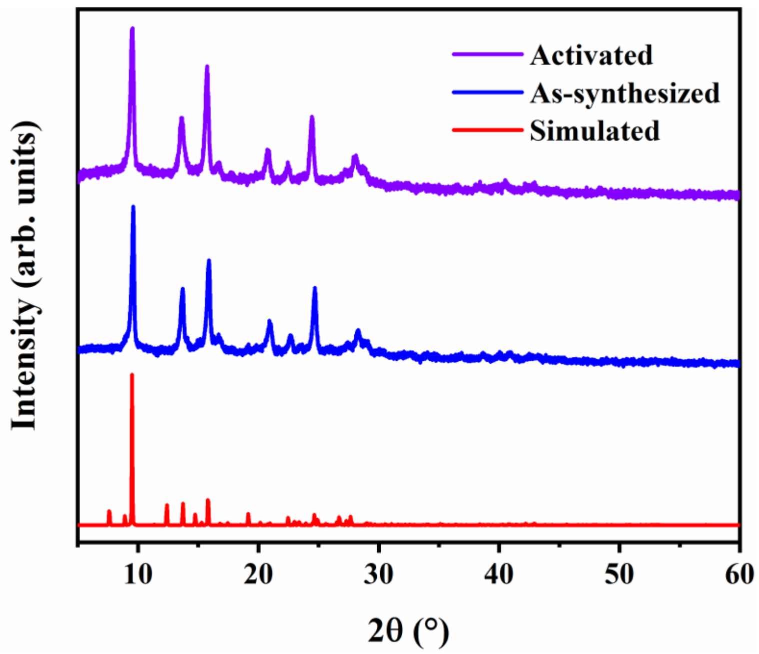

Supplementary Figure 3. PXRD patterns data. PXRD patterns of simulated, as-synthesized, and activated SOFOUR-DPDS-Ni.

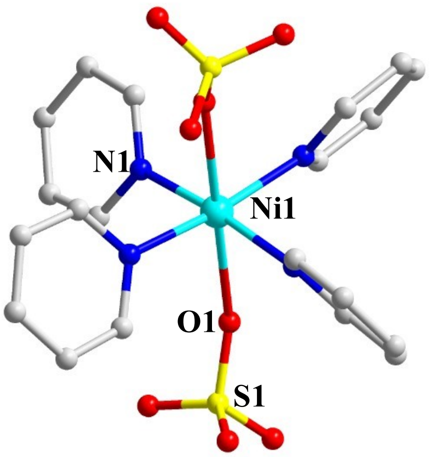

Supplementary Figure 4. Coordination mode. The coordination environment of SOFOUR-DPDS-Ni. The rest of 4-DPDS ligands and hydrogen atoms are omitted for clarity. Cyan = Ni, grey = C, red = O, blue = N, yellow = S. Selected bond lengths: Ni1-N1 = 2.044 Å, Ni1-O1 = 2.476 Å. Selected bond angles: Ni1-O1-S1 = 150.556°.

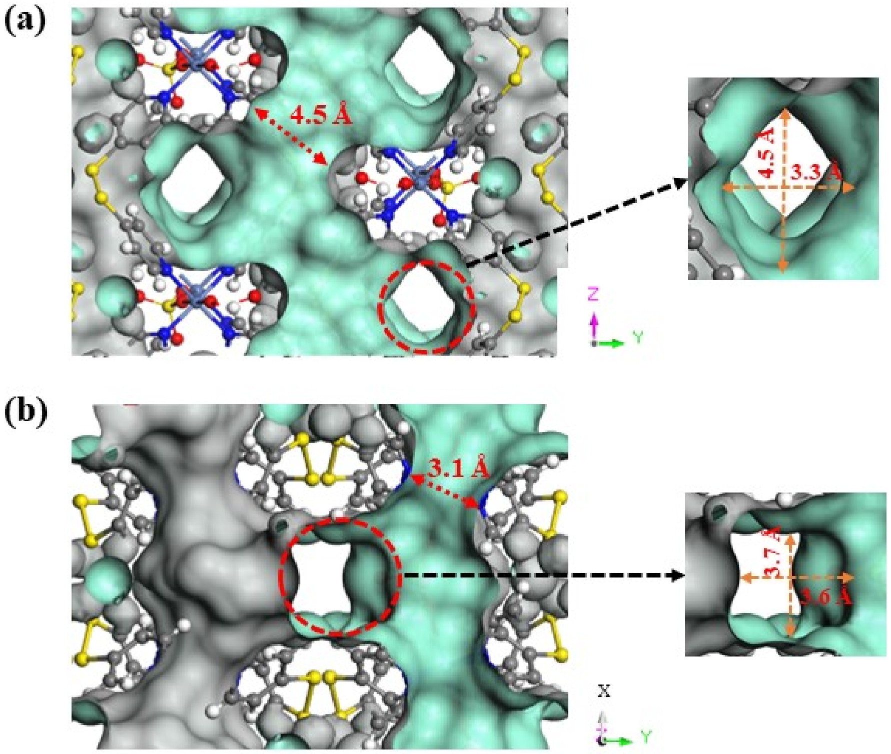

Supplementary Figure 5. Connolly surface. Accessible Connolly surface of SOFOUR-DPDS-Ni along the (a) *X-axes* and (b) *Z-axes* determined by using a probe with the radius of 1.0 Å.

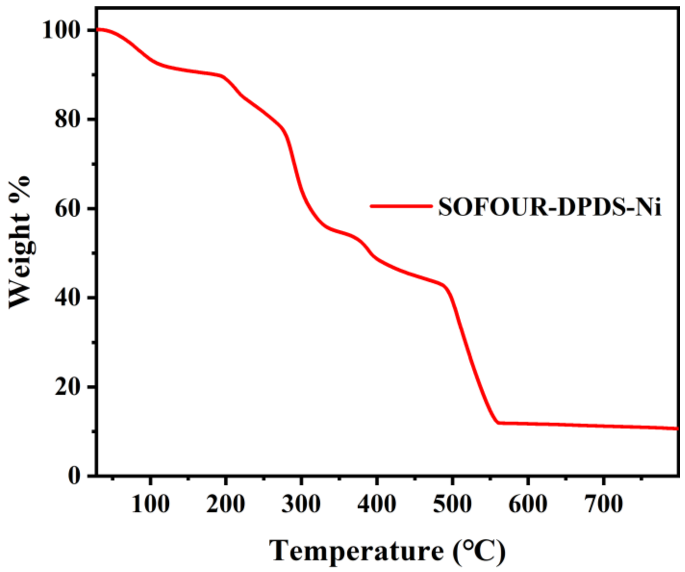

Supplementary Figure 6. Thermal stability. Thermogravimetric analysis curves of as-synthesized SOFOUR-DPDS-Ni.

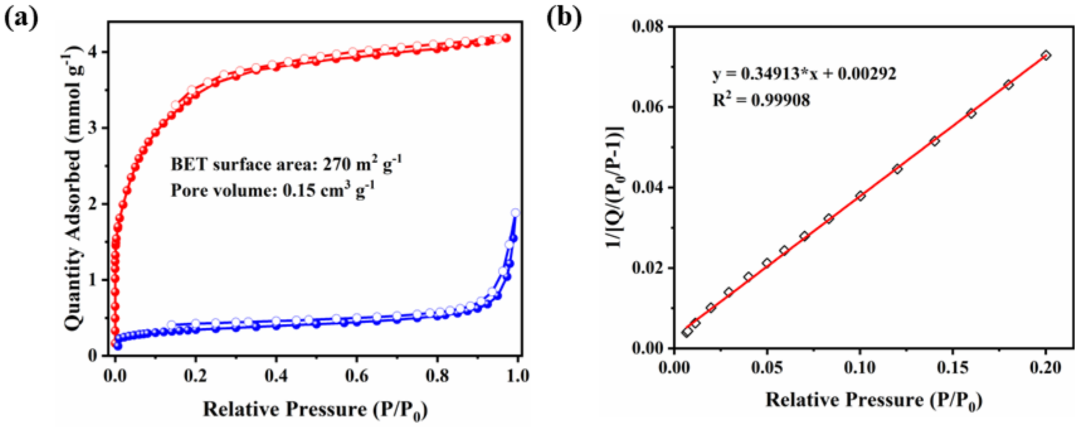

Supplementary Figure 7. Pore structure property. (a). Low-temperature CO_2 (195 K) and N_2 (77 K) sorption isotherms of SOFOUR-DPDS-Ni; (b). Calculation plot of BET surface area based on corresponding CO_2 adsorption isotherms of SOFOUR-DPDS-Ni at 195 K.

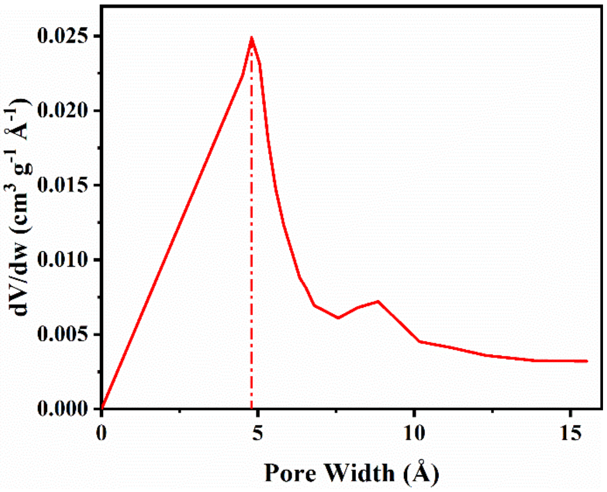

Supplementary Figure 8. Pore width distribution. Pore width distribution for SOFOUR-DPDS-Ni based on Horvath-Kawazoe model determined by CO_2 adsorption isotherms at 195 K.

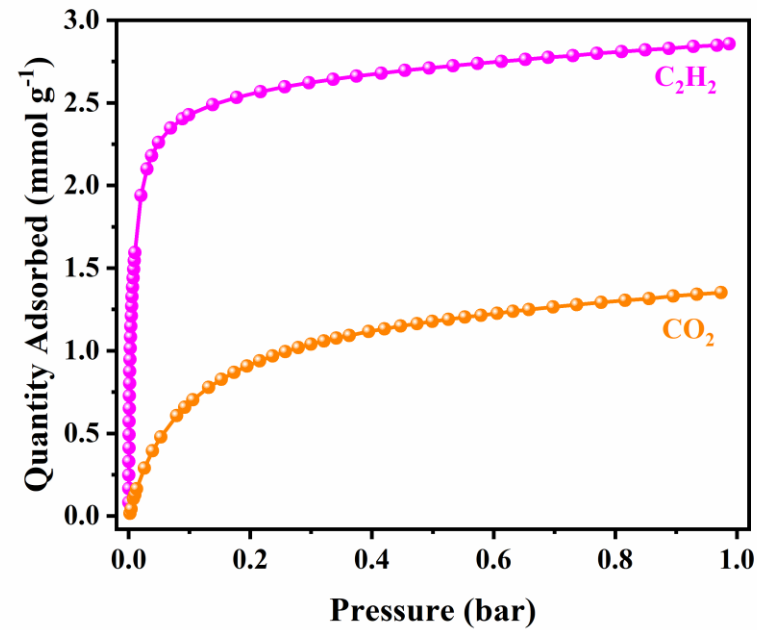

Supplementary Figure 9. Adsorption isotherms. Adsorption isotherms of C_2H_2 and CO_2 on SOFOUR-DPDS-Ni at 298 K.

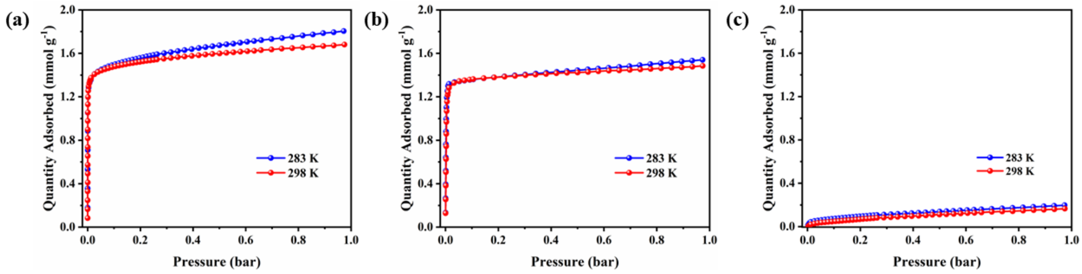

Supplementary Figure 10. Adsorption isotherms. Adsorption isotherms of (a) C_4H_6, (b) n-C_4H_8, and (c) iso-C_4H_8 on SOFOUR-DPDS-Ni at 283 K and 298 K.

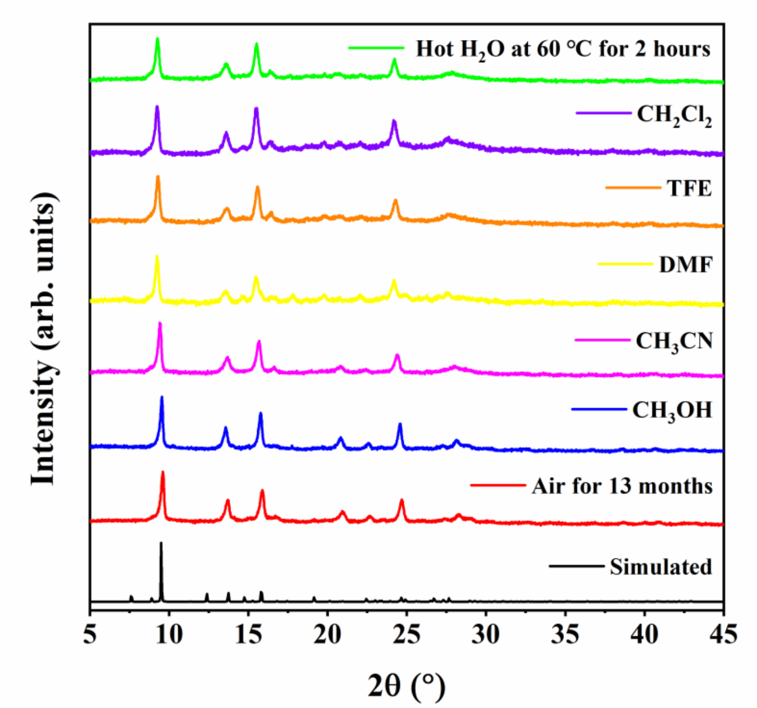

Supplementary Figure 11. Stability tests. PXRD patterns of SOFOUR-DPDS-Ni after different treating methods.

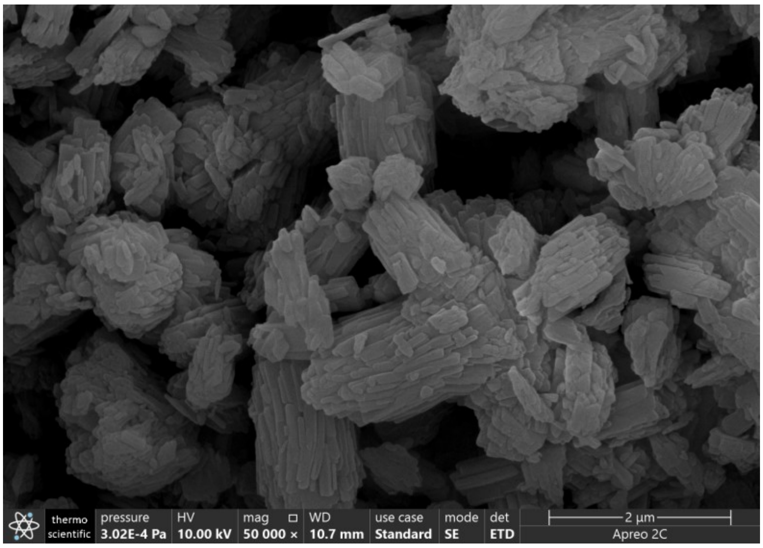

Supplementary Figure 12. SEM image. SEM image of SOFOUR-DPDS-Ni.

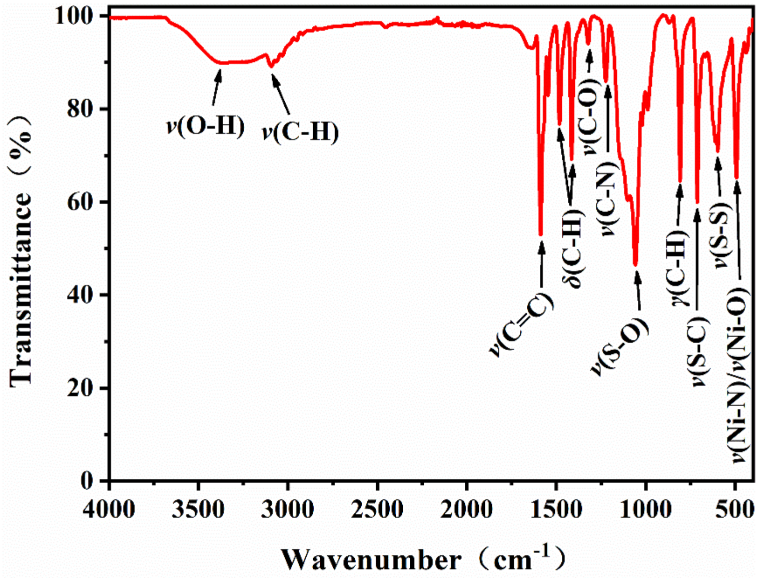

Supplementary Figure 13. FT-IR spectrum. The FT-IR spectrum of SOFOUR-DPDS-Ni.

The broad stretching vibration of the -OH group at 3372.0 cm^{-1} was attributed to the presence of CH_3OH molecules in the framework. The strong peak at 1588.1 cm^{-1} corresponded to the stretching vibration of C=C in the pyridine ring. The characteristic peaks at 3093.3, 1482.5, and 1415.5 cm^{-1} represented the bending vibration of C-H bonds in either CH_3OH molecules or pyridine rings. The weak peak at 1323.0 cm^{-1} indicated the stretching vibration of C-O in CH_3OH molecules. The weak peak located at 1223.3 cm^{-1} belonged to the stretching vibration of C-N in the pyridine ring. The peak at 1058.3 cm^{-1} represented the antisymmetric stretching vibration of S-O in the SO_4^{2-} group. The peaks at 712.1 and 595.9 cm^{-1} were assigned to the stretching vibrations of S-C and S-S, respectively. The peak at 493.2 cm^{-1} corresponded to the Ni-O or Ni-N stretching vibration.

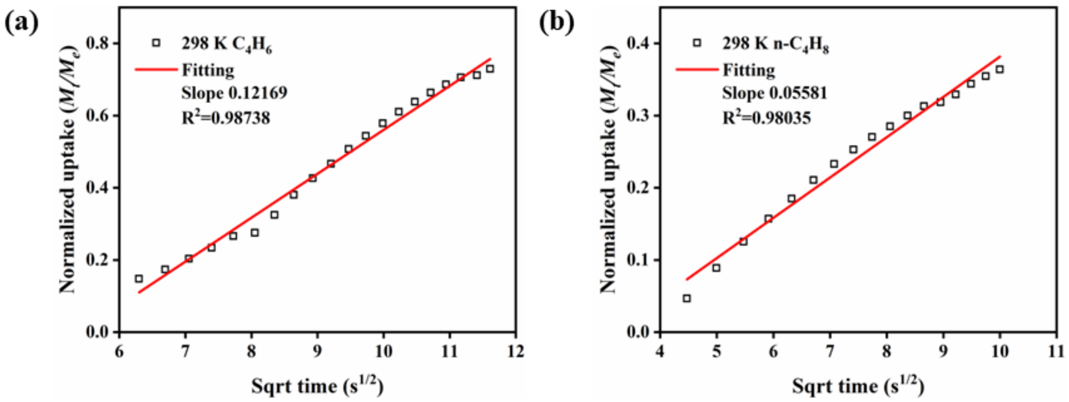

Supplementary Figure 14. Fitting of kinetic curve. Kinetic profile of (a) C_4H_6 and (b) n-C_4H_8 on SOFOUR-DPDS-Ni.

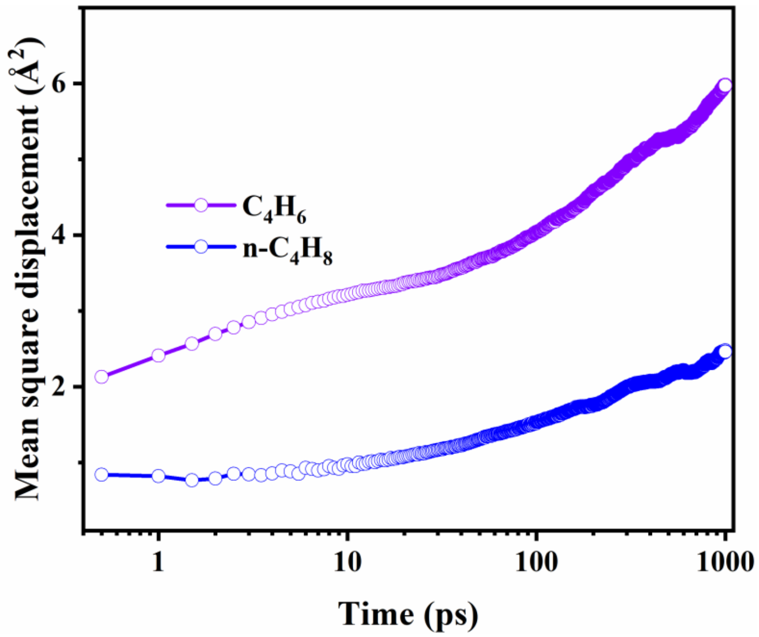

Supplementary Figure 15. MSD. Mean-squared displacements of C_4H_6 and n-C_4H_8 on SOFOUR-DPDS-Ni at 298 K.

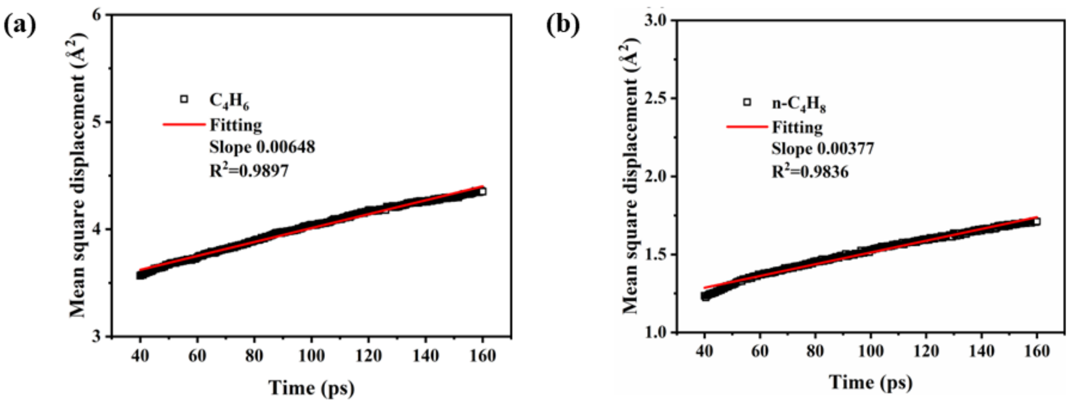

Supplementary Figure 16. Fitting of MSD. Self-diffusion coefficients of (a) C_4H_6 and (b) n-C_4H_8 in SOFOUR-DPDS-Ni at 298 K.

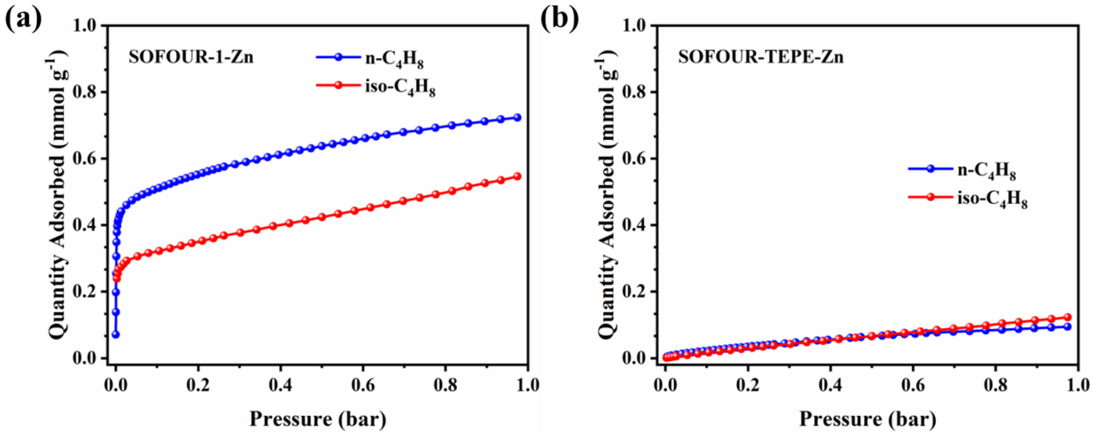

Supplementary Figure 17. Adsorption isotherms. Adsorption isotherms of C_4 olefins on (a) SOFOUR-1-Zn and (b) SOFOUR-TEPE-Zn at 298 K.

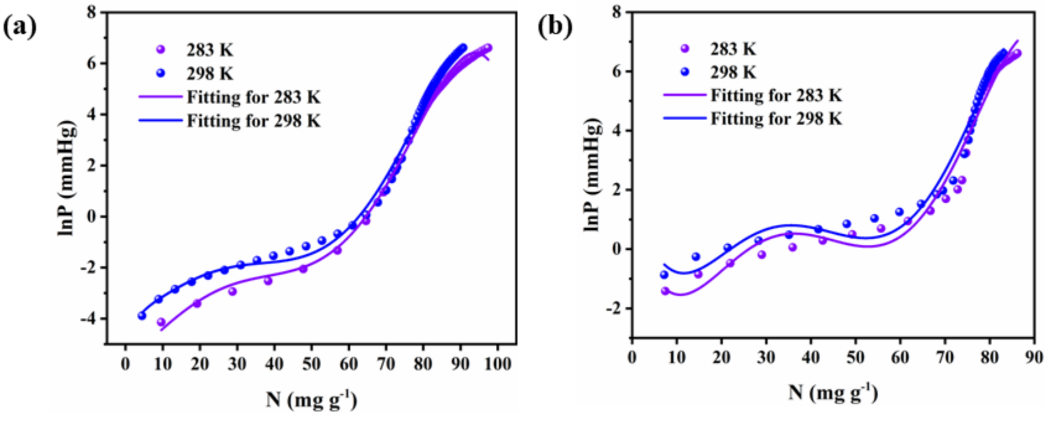

Supplementary Figure 18. Virial fitting. The virial fitting of (a) C_4H_6 and (b) n-C_4H_8 adsorption isotherms for SOFOUR-DPDS-Ni.

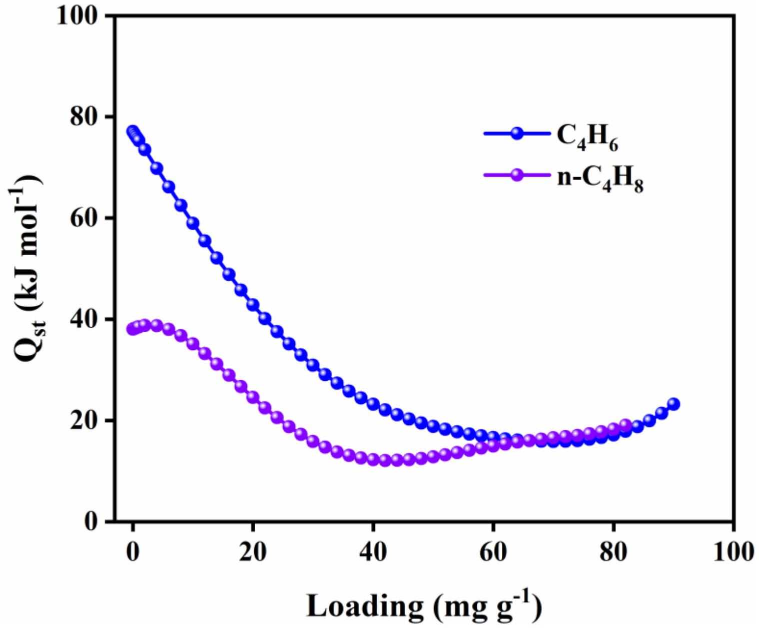

Supplementary Figure 19. Adsorption heat curves. The isosteric heats of adsorption (Q_{st}) of C_4H_6 and n-C_4H_8 on SOFOUR-DPDS-Ni.

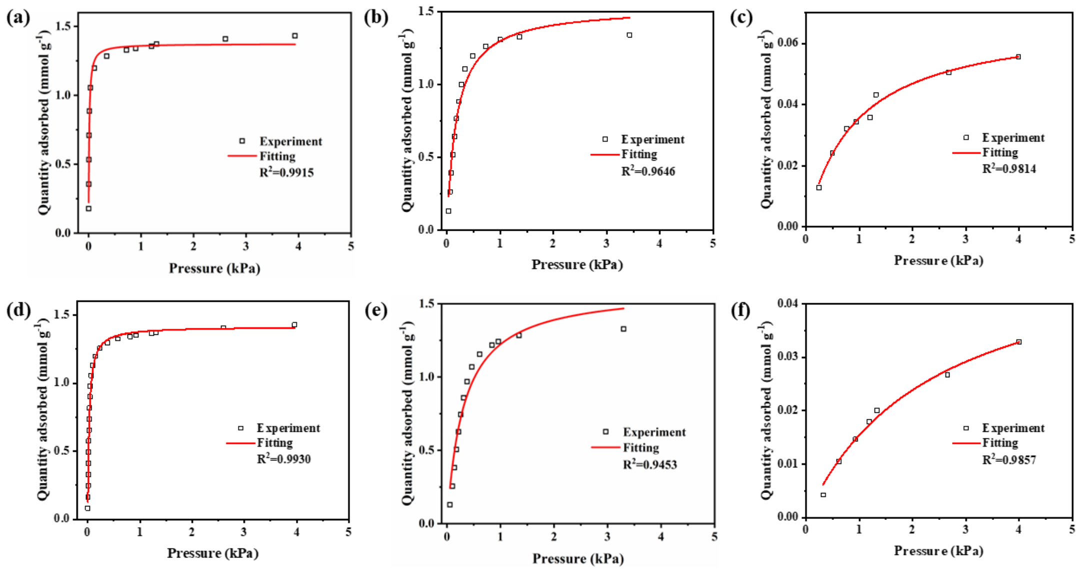

Supplementary Figure 20. Langmuir fitting curves. The Langmuir fittings of C_4H_6, n-C_4H_8, and iso-C_4H_8 isotherms at 0-5 kPa and (a, b, c) 283 K and (d, e, f) 298K.

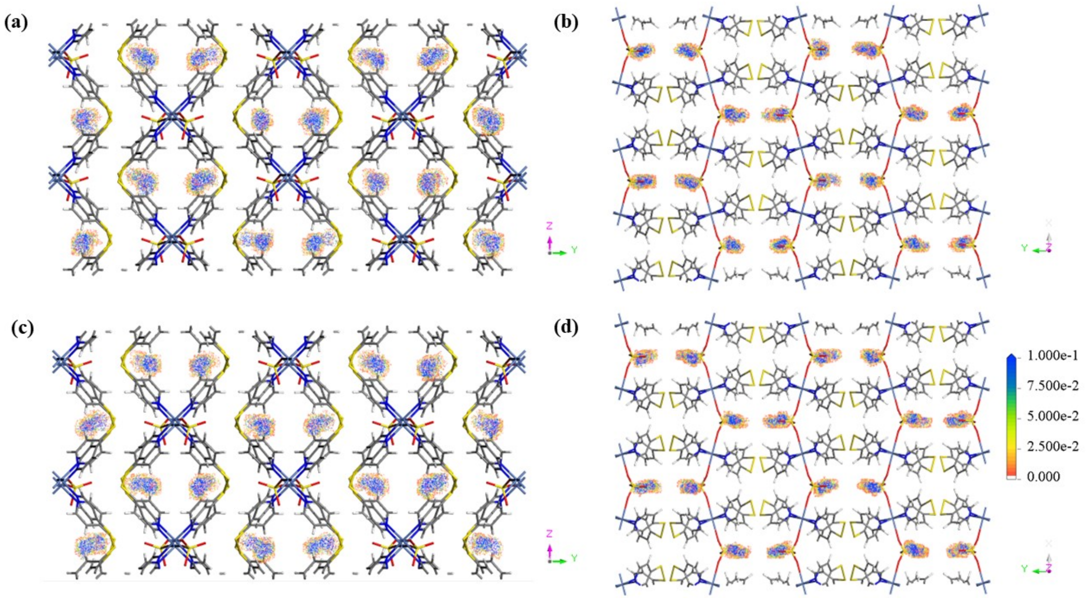

Supplementary Figure 21. GCMC simulated curves. GCMC simulated C_4H_6 adsorption in SOFOUR-DPDS-Ni at (a,b) 0.01 and (c,d) 1.0 bar.

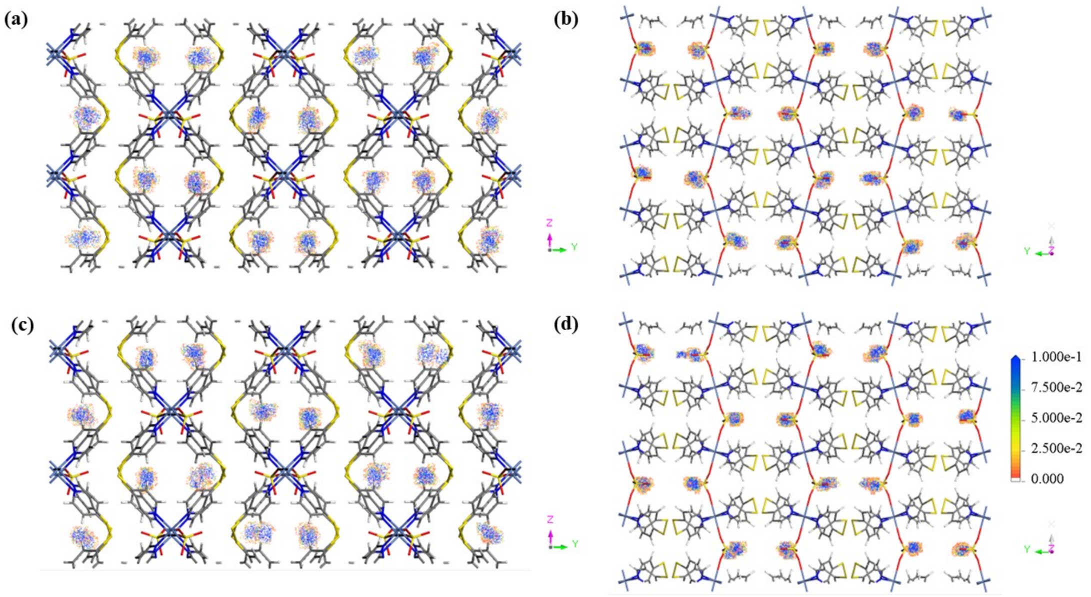

Supplementary Figure 22. GCMC simulated curves. GCMC simulated n-C_4H_8 adsorption in SOFOUR-DPDS-Ni at (a,b) 0.01 and (c,d) 1.0 bar.

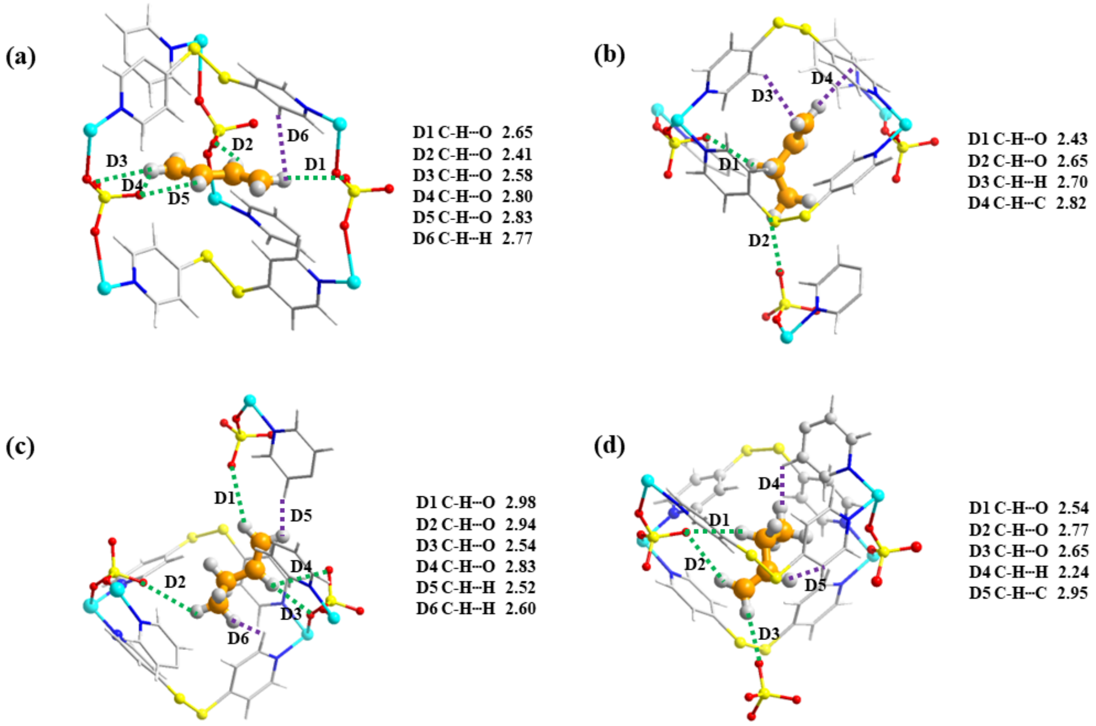

Supplementary Figure 23. Binding sites. DFT calculated adsorption binding site for (a) C_4H_6 and (b, c, d) n-C_4H_8 in SOFOUR-DPDS-Ni.

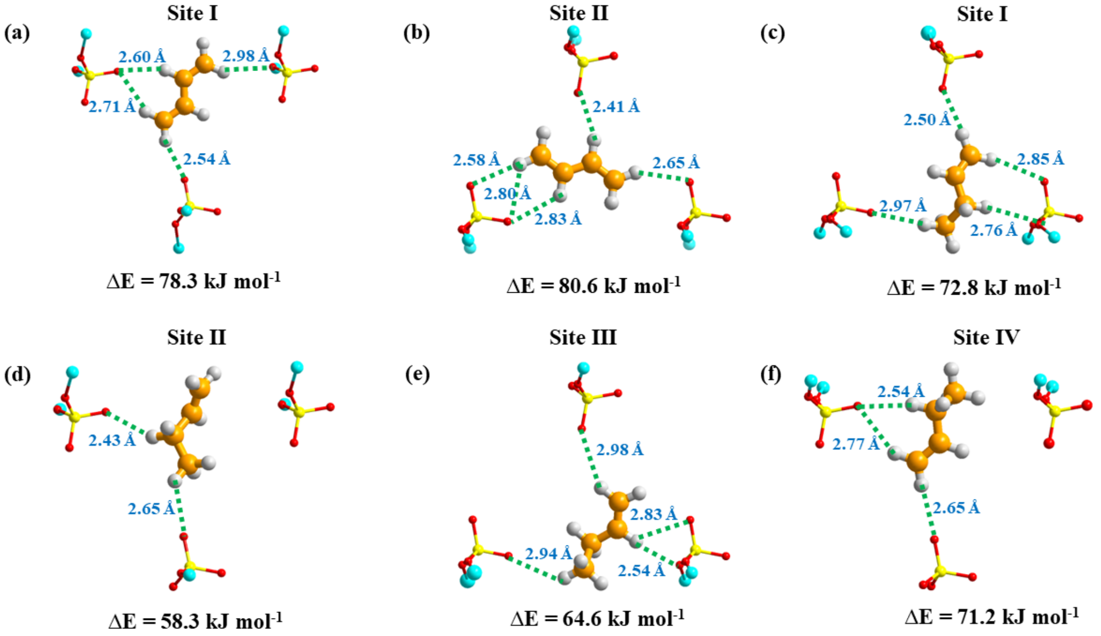

Supplementary Figure 24. Binding energy. DFT calculated C-H···O interactions and binding energy of (a-b) C_4H_6 and (c-f) n-C_4H_8.

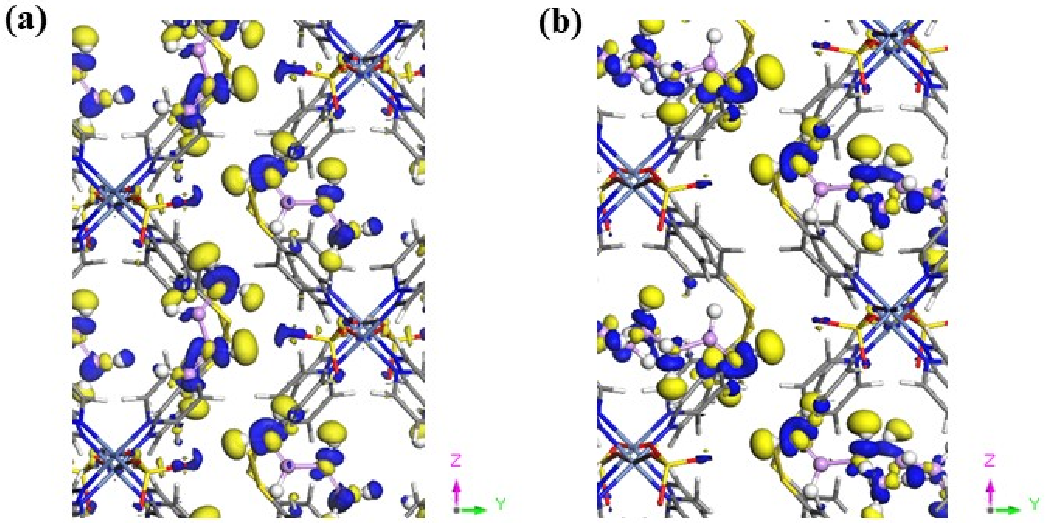

Supplementary Figure 25. Charge density difference diagram. Charge density difference plots between SOFOUR-DPDS-Ni framework and (a) C_4H_6 and (b) n-C_4H_8.

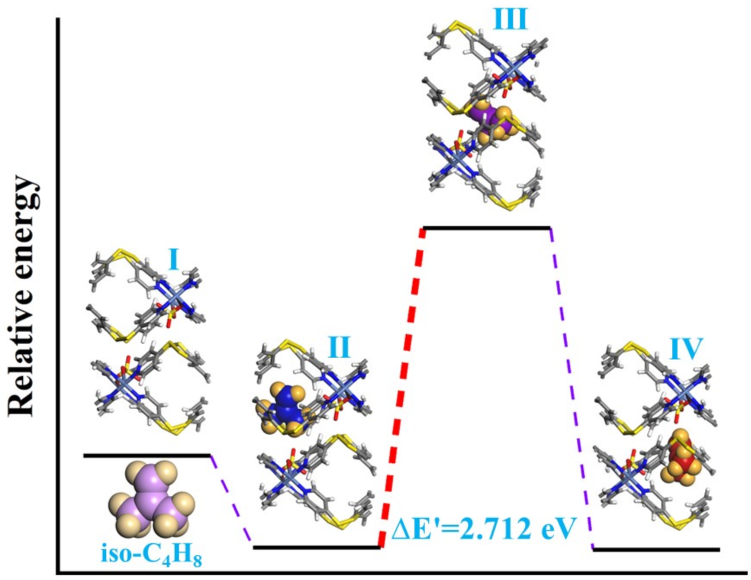

Supplementary Figure 26. Energy barrier. Illustration of the interaction energy pathway and the corresponding energy levels calculated by DFT for iso-C_4H_8.

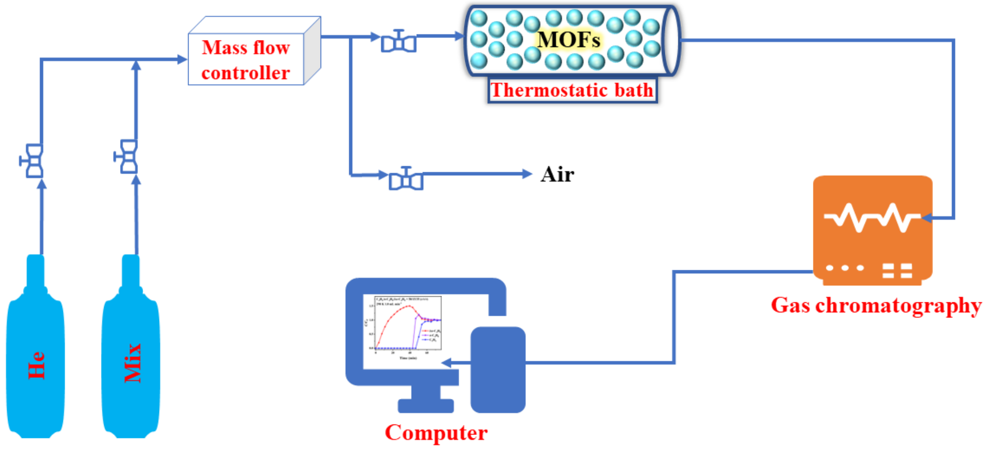

Supplementary Figure 27. Experimental devise. Schematic illustration of the apparatus for the breakthrough experiments.

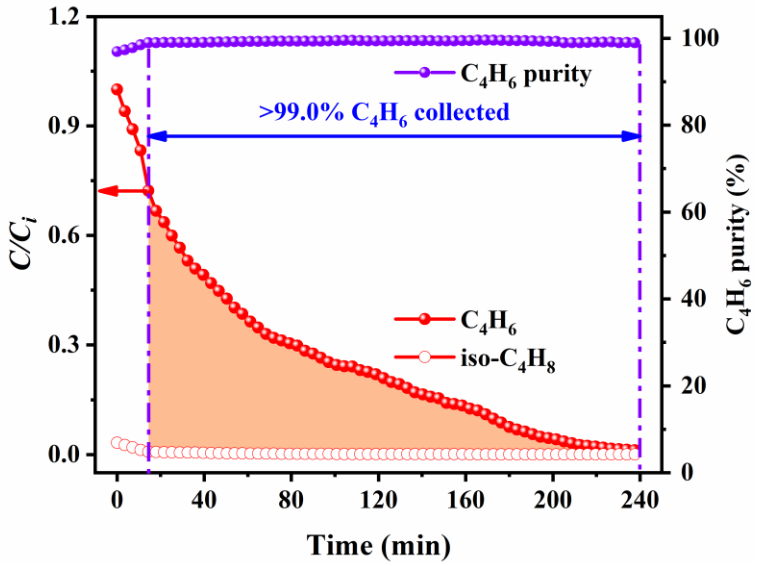

Supplementary Figure 28. Desorption experiment. Desorption curve for C_4H_6/iso-C_4H_8 breakthrough experiment on SOFOUR-DPDS-Ni.

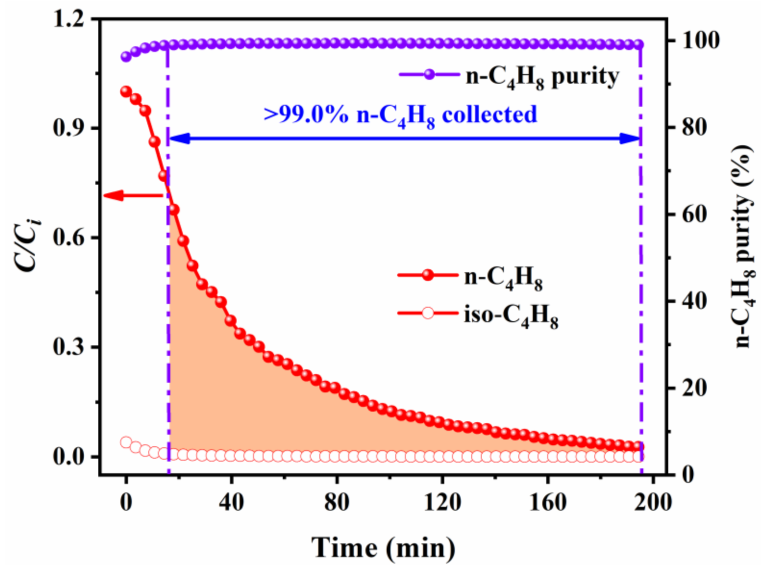

Supplementary Figure 29. Desorption experiment. Desorption curve for n-C_4H_8/iso-C_4H_8 breakthrough experiment on SOFOUR-DPDS-Ni.

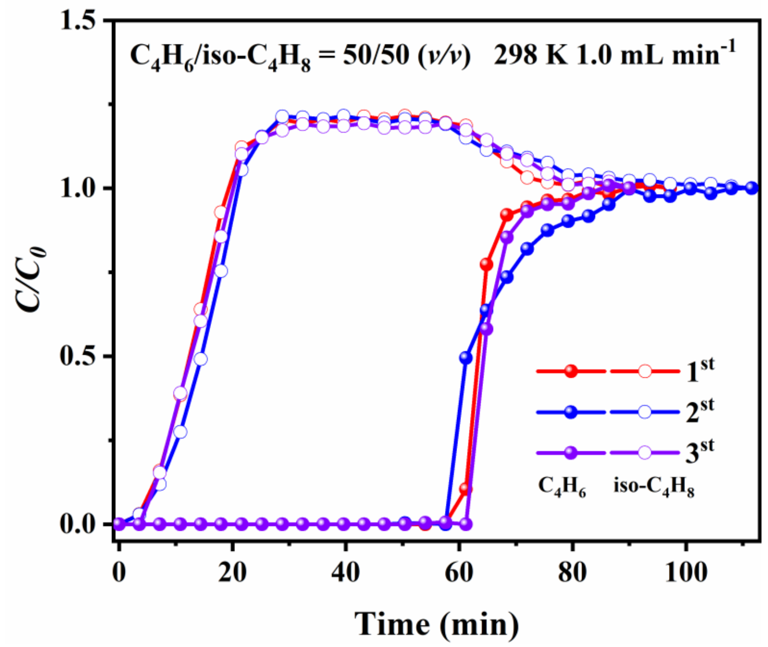

Supplementary Figure 30. Dynamic breakthrough curves. Cycling dynamic breakthrough tests for C_4H_6/iso-C_4H_8 (50/50, *v/v*) under the flow rate of 1.0 mL min^{-1}.

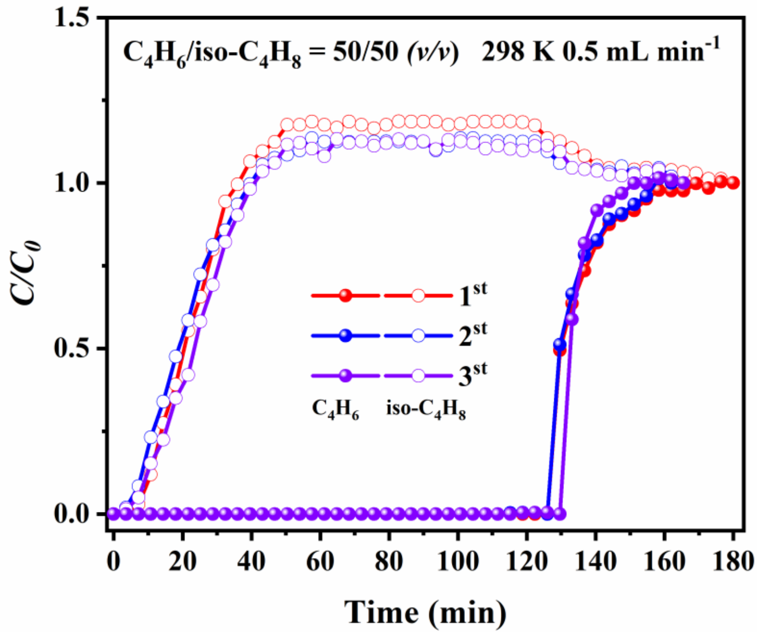

Supplementary Figure 31. Dynamic breakthrough curves. Cycling dynamic breakthrough tests for C_4H_6/iso-C_4H_8 (50/50, *v/v*) under the flow rate of 0.5 mL min^{-1}.

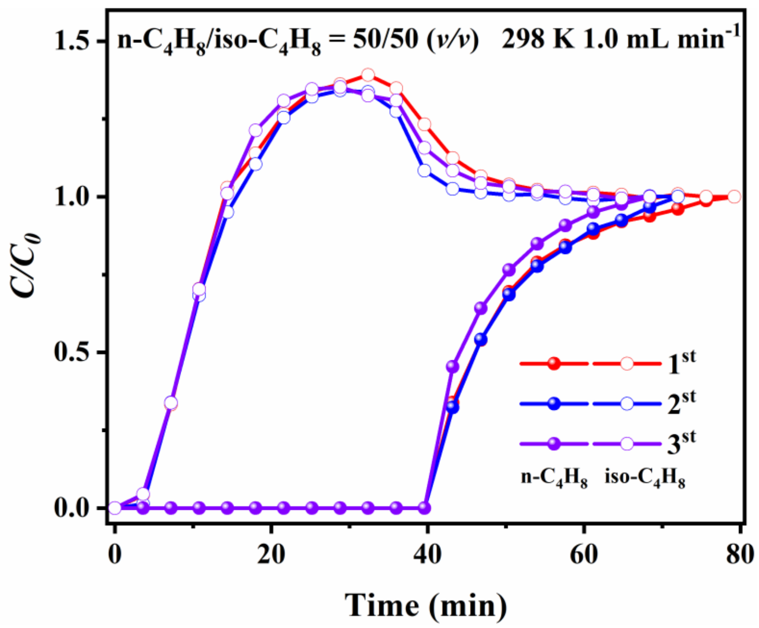

Supplementary Figure 32. Dynamic breakthrough curves. Cycling dynamic breakthrough tests for n-C_4H_8/iso-C_4H_8 (50/50, *v/v*) under the flow rate of 1.0 mL min^{-1}.

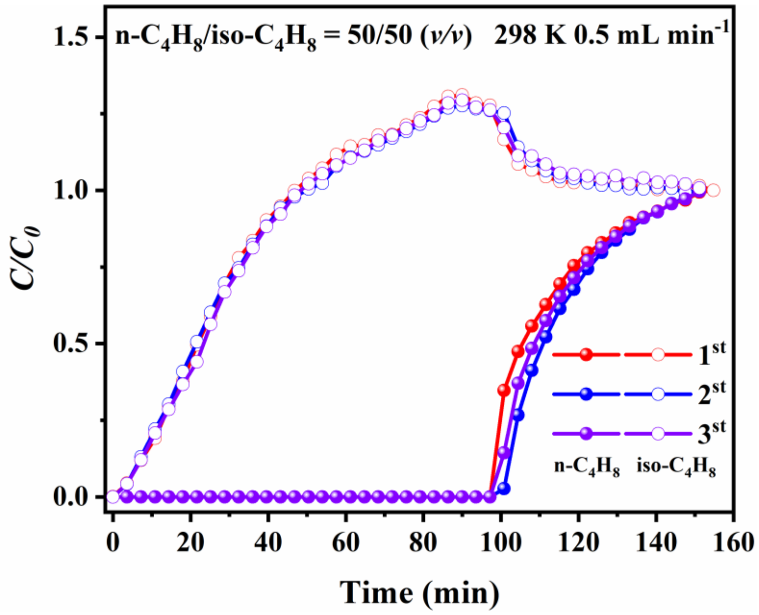

Supplementary Figure 33. Dynamic breakthrough curves. Cycling dynamic breakthrough tests for n-C_4H_8/iso-C_4H_8 (50/50, *v/v*) under the flow rate of 0.5 mL min^{-1}.

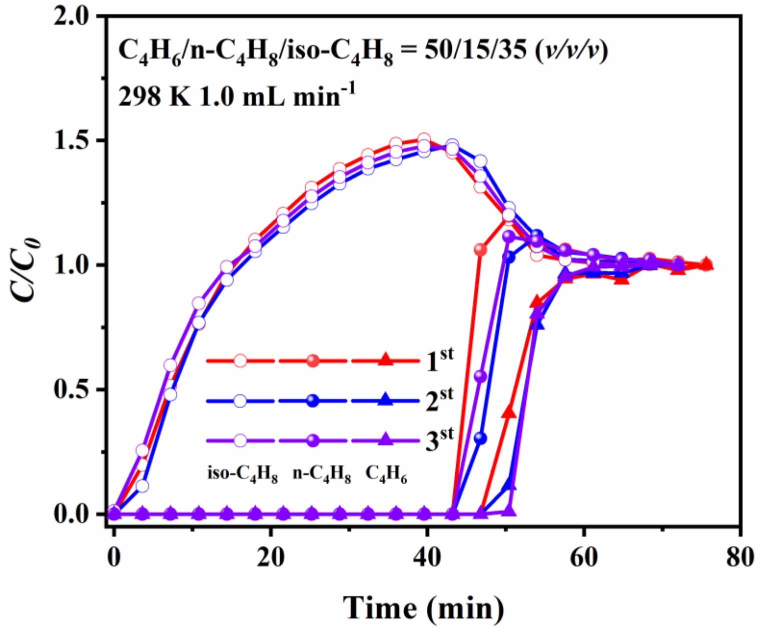

Supplementary Figure 34. Dynamic breakthrough curves. Cycling dynamic breakthrough tests for C_4H_6/n-C_4H_8/iso-C_4H_8 (50/15/50, *v/v/v*) under the flow rate of 1.0 mL min^{-1}.

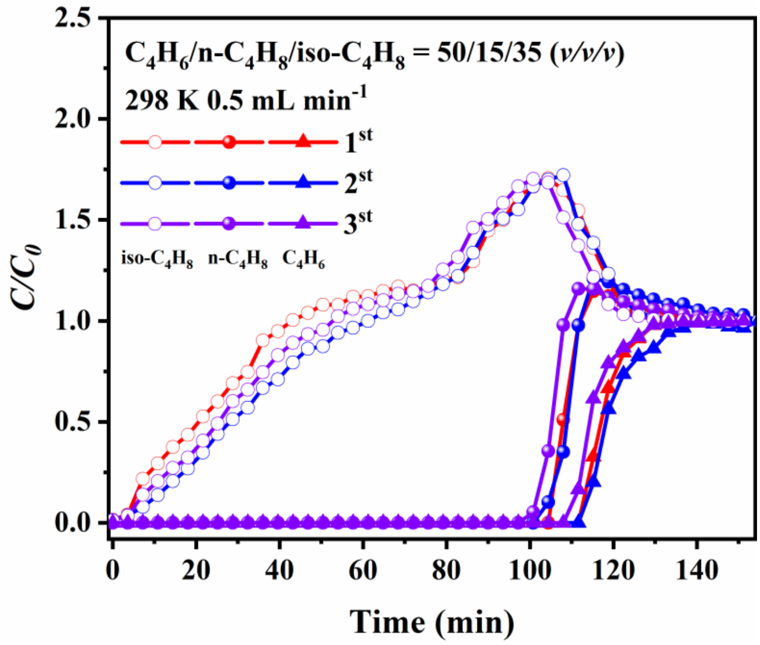

Supplementary Figure 35. Dynamic breakthrough curves. Cycling dynamic breakthrough tests for C_4H_6/n-C_4H_8/iso-C_4H_8 (50/15/50, *v/v/v*) under the flow rate of 0.5 mL min^{-1}.

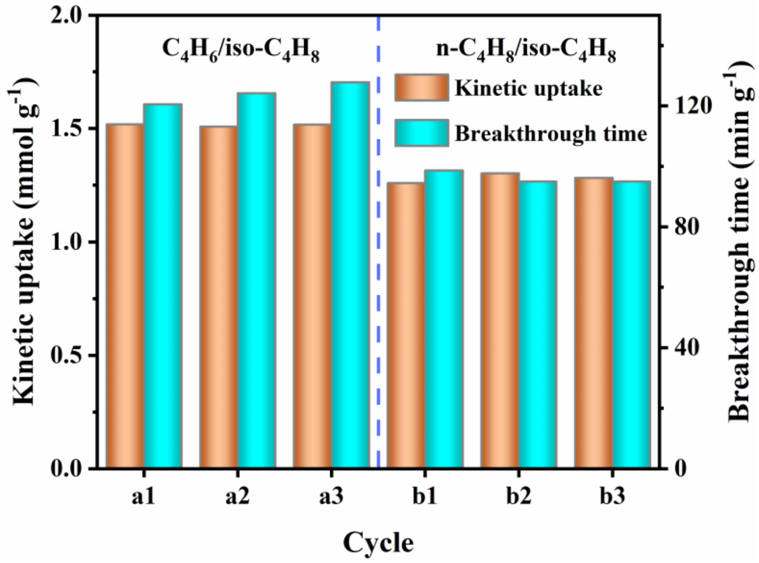

Supplementary Figure 36. Dynamic breakthrough experiments. Cycling breakthrough tests for C_4H_6/iso-C_4H_8 (a1-a3) and n-C_4H_8/iso-C_4H_8 (b1-b3) separation under the flow rate of 0.5 mL min^{-1}.

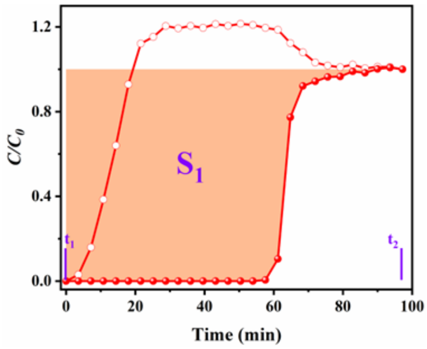

The kinetic adsorption capacity (Q) of C_4H_6 or n-C_4H_8 is calculated as:

$$Q = \frac{v \times V\%}{m \times 22.4} \times \int_{t_1}^{t_2} (c_1 - c_i)dt = \frac{v \times V\%}{m \times 22.4} \times S_1$$

v is the flow rate of the gas mixture, $V\%$ is the molar fraction of C_4H_6 or n-C_4H_8, and m is the mass of the adsorbent.

Supplementary Figure 37. Schematic diagram of calculation. The calculation diagram of kinetic adsorption capacity of C_4H_6 or n-C_4H_8.

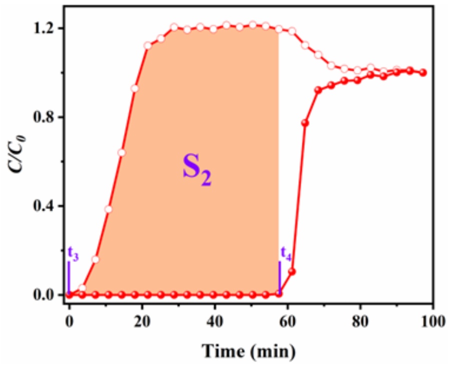

The productivity (q) of iso-C_4H_8 is calculated as:

$$q = \frac{v \times V\%}{m \times 22.4} \times \int_{t_3}^{t_4} (c_x - c_i)dt = \frac{v \times V\%}{m \times 22.4} \times S_2$$

v is the flow rate of the gas mixture, $V\%$ is the molar fraction of iso-C_4H_8, and m is the mass of the adsorbent.

Supplementary Figure 38. Schematic diagram of calculation. The calculation diagram of iso-C_4H_8 productivity.

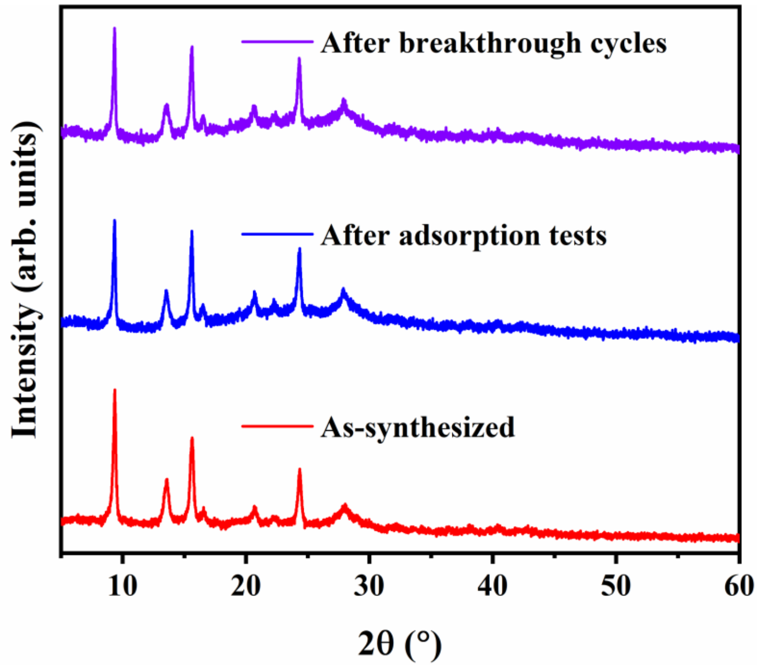

Supplementary Figure 39. Crystallinity and integrity. PXRD patterns of SOFOUR-DPDS-Ni after cycled adsorption tests and breakthrough experiments.

Supplementary Table 1. Ultimate element analysis of as-synthesized SOFOUR-DPDS-Ni.

Elemental		C	H	N	S	N/S
mass	Measured	36.82	3.10	8.47	21.56	0.39
(wt. %)	Theoretical	40.34	2.69	9.41	26.93	0.35

Supplementary Table 2. Physical properties of C_4H_6/n-C_4H_8/iso-C_4H_8.

Compound	Molecular dimension (Å)			Kinetic diameter (Å)	Polarizability (10^{-25} cm^3)	Boiling point (K)
	X	Y	Z			
C_4H_6	3.40	5.36	7.84	4.31	86	268.6
n-C_4H_8	4.05	4.17	7.87	4.46	81	266.9
iso-C_4H_8	4.16	5.76	6.71	4.84	80	266.3

Supplementary Table 3. Langmuir fit parameters for C_4H_6, n-C_4H_8, and iso-C_4H_8 in SOFOUR-DPDS-Ni at 283 K and 0-5 kPa.

Adsorbates	q_m (mmol g^{-1})	b (kPa^{-1})	q_m×b	R^2
C_4H_6	1.3739	91.0705	125.1218	0.9915
n-C_4H_8	1.5267	5.5275	8.4388	0.9646
iso-C_4H_8	0.06848	1.0883	0.07453	0.9814

Supplementary Table 4. Langmuir fit parameters for C_4H_6, n-C_4H_8, and iso-C_4H_8 in SOFOUR-DPDS-Ni at 298 K and 0-5 kPa.

Adsorbates	q_m (mmol g^{-1})	b (kPa^{-1})	q_m×b	R^2
C_4H_6	1.4158	35.9302	50.8700	0.9930
n-C_4H_8	1.6074	3.1821	5.1149	0.9453
iso-C_4H_8	0.05244	0.4179	0.02191	0.9857

Supplementary Table 5. The virial parameters for calculated Q_{st} of C_4H_6 and n-C_4H_8 on SOFOUR-DPDS-Ni at 283 K and 298 K up to 1.0 bar.

Virial coefficient	C_4H_6	n-C_4H_8
	Value	Value
a_0	-9273.083	-4572.492
a_1	215.8144	-74.19575
a_2	1.355563	15.00305
a_3	-0.1237129	-0.4628355
a_4	0.001721873	0.005482776
a_5	-7.910118E-6	-2.298421E-5
b_0	26.32418	16.83510
b_1	-0.8772939	-0.6417600
b_2	0.007916788	0.006767006
R^2	0.9962	0.9780
Equation	$y = \ln(x)+1/T*(a_0+a_1*x+a_2*x^2+a_3*x^3+a_4*x^4+a_5*x^5) + (b_0+b_1*x+b_2*x^2)$	

Supplementary Table 6. Comparison of separation selectivity based on uptake ratio of reported materials.

Materials	Temperature (K)	Pressure (kPa)	Uptake selectivity			Reference
			C_4H_6/n-C_4H_8	C_4H_6/iso-C_4H_8	n-C_4H_8/iso-C_4H_8	
SOFOUR-DPDS-Ni	298	100	1.1	9.9	8.7	This work
TMOF-1	298	100	1.5	8.2	5.4	1
ZU-619	298	100	2.51	4.65	1.85	1
Mn-bpdc	298	101	40	45	1.1	2
Y-fum-**fcu**-MOF	303	101	0.97	2.66	2.76	3
Mg-gallate	298	101	1.3	15.1	11.2	4
Co-gallate	298	101	2.1	14.3	6.8	4
Ni-gallate	298	101	2.4	15.4	6.3	4
SIFSIX-1-Cu	298	101	1.1	1.1	1.0	5
SIFSIX-3-Ni	298	101	1.0	4.7	4.7	5
GeFSIX-14-Cu-i	298	101	4.7	6.4	1.4	5
NbFSIX-2-Cu-i	298	101	1.2	5.7	5.0	5
GeFSIX-2-Cu-i	298	101	1.1	2.9	2.3	5
SIFSIX-2-Cu-I	298	101	1.3	2.3	1.8	5
TIFSIX-2-Cu-i	298	101	1.1	1.4	1.3	5
ZJNU-30a	298	100	1.0	1.2	1.2	6
ZJNU-80a	298	101	1.0	1.2	1.2	7
NOTT-101a	298	101	1.0	1.0	1.0	7
SD-65	298	101	22.3	25.4	1.1	8
Zn(Hmpba)$_2$	303	100	1.1	1.1	1	9
Mg-DOBDC	-	-	1.0	1.0	1.0	10
Co-DOBDC	-	-	1.1	1.1	1.0	10
Zn-EIM-RHO	-	-	1.0	1.0	1.0	10
SC-K	298	100	3.75	4.72	1.26	11
ZU-36-Co	298	101	-	-	13.8	12

Supplementary Table 7. Comparison of Henry's selectivity (α_{ij}) of various materials.

Materials	Temperature (K)	Pressure (kPa)	$\alpha_{C4H6/n-C4H8}$	$\alpha_{C4H6/iso-C4H8}$	$\alpha_{n-C4H8/iso-C4H8}$	reference
SOFOUR-DPDS-Ni	298	100	9.94	2321.8	233.5	This work
TMOF-1	298	100	-	519.2	93.2	1
ZU-619	298	100	-	72.58	6.36	1
Mg-gallate	298	101	3.7	106	29	4
Co-gallate	298	101	1.6	63	40	4
Ni-gallate	298	101	2.2	148	67	4
SIFSIX-1-Cu	298	101	0.5	1.6	3.4	5
GeFSIX-14-Cu-i	298	101	95.3	228.7	2.4	5
NbFSIX-2-Cu-i	298	101	1.5	75.2	48.9	5
GeFSIX-2-Cu-i	298	101	4.0	5.4	1.4	5
SIFSIX-2-Cu-i	298	101	3.2	4.6	1.5	5
SD-65	298	101	3.4	3.7	1.1	8
$Zn(Hmpba)_2$	303	100	1.4	3.0	2.1	9

Supplementary Table 8. PXRD Rietveld refinements of the modeled structure of SOFOUR-DPDS-Ni.

Unit cell parameters	SOFOUR-DPDS-Ni
Formular	$C_{80}H_{64}N_{16}O_{16}S_{20}Ni_4$
Formula weight	2381.42
Cell volume	2987.08
Crystal system	Orthorhombic
Space group	Pnna (52)
a (Å)	10.53768
b (Å)	14.29568
c (Å)	19.82885
α (°)	90.000
β (°)	90.000
γ (°)	90.000
D_{calcd} (g cm^{-3})	1.32390
R_p[a]	0.0399
R_{wp}[b]	0.0561

$aR_p = \sum|cY_{sim}(2\theta i) - I_{exp}(2\theta i) + Y_{back}(2\theta i)|/\sum|I_{exp}(2\theta i)|$.

$bR_{wp} = \{w_p[cY_{sim}(2\theta i) - I_{exp}(2\theta i) + Y_{back}(2\theta i)]_2/\sum w_p[I_{exp}(2\theta i)]_2\}_{1/2}$, $w_p = 1/I_{exp}(2\theta i)$.

Supplementary Table 9. List of atomic coordinates for the modeled structure of SOFOUR-DPDS-Ni.

Atoms	X	Y	Z	B	Site
S	-0.20820	0.50020	0.18810	0.07397	0.50
S	0.21720	0.22130	0.01630	0.09485	1.00
S	0.33290	0.11030	0.03640	0.09636	1.00
O	-0.22720	0.41750	0.21830	0.10586	1.00
O	-0.07000	0.51260	0.20570	0.14279	0.50
N	-0.09110	0.23010	0.17730	0.07069	1.00
N	0.63880	0.21130	0.18130	0.06330	1.00
C	0.00430	0.16840	0.18360	0.08182	1.00
H	0.00460	0.12910	0.22140	0.09720	1.00
H	0.16910	0.11480	0.14360	0.09240	1.00
H	0.38410	0.29080	0.10590	0.08160	1.00
H	0.54680	0.33490	0.17850	0.08400	1.00
H	0.70240	0.08050	0.16740	0.08880	1.00
H	0.54280	0.03020	0.09350	0.08640	1.00
H	-0.00200	0.32320	0.03290	0.09600	1.00
H	-0.15920	0.33430	0.11690	0.09280	1.00
C	0.10170	0.15960	0.13750	0.07787	1.00
C	0.09920	0.21790	0.08130	0.07272	1.00
C	0.44910	0.15510	0.09320	0.06794	1.00
C	0.45080	0.24430	0.11920	0.06873	1.00
C	0.54700	0.26910	0.16220	0.07068	1.00
C	0.63610	0.12420	0.15470	0.07466	1.00
C	0.54320	0.09360	0.11130	0.07261	1.00
C	0.00250	0.28200	0.07310	0.08079	1.00
C	-0.09020	0.28520	0.12260	0.07805	1.00
Ni	-0.22600	0.25000	0.25000	0.07821	1.00
O	-0.25000	0.50000	0.11990	0.27652	1.00

Supplementary References

1. Cui, J. *et al.* Geometry control of adsorption sites in sulfonate-pillared hybrid ultramicroporous materials for efficient C4 olefin separations. *Chem. Eng. J.* **425**, 130580 (2021).
2. Lu, W. *et al.* Efficient separation of 1,3-butadiene from C4 hydrocarbons by flexible metal–organic framework with gate-opening effect. *AIChE J.* **68**, e17568 (2022).
3. Assen, A. H. *et al.* Kinetic separation of C4 olefins using Y-fum-fcu-MOF with ultra-fine-tuned aperture size. *Chem. Eng. J.* **413**, 127388 (2021).
4. Chen, J. *et al.* Adsorptive separation of geometric isomers of 2-butene on gallate-based metal–organic frameworks. *ACS Appl. Mater. Interfaces* **12**, 9609–9616 (2020).
5. Zhang, Z. *et al.* Sorting of C_4 olefins with interpenetrated hybrid ultramicroporous materials by combining molecular recognition and size-sieving. *Angew. Chem. Int. Ed.* **56**, 16282–16287 (2017).
6. Liu, H. *et al.* A porous zirconium-based metal-organic framework with the potential for the separation of butene isomers. *Chem. Eur. J.* **22**, 14988–14997 (2016).
7. Jiao, J., Liu, H., Bai, D. & He, Y. A chemically cross-linked NbO-Type metal–organic framework: cage or window partition. *Inorg. Chem.* **55**, 3974–3979 (2016).
8. Kishida, K. *et al.* Recognition of 1,3-butadiene by a porous coordination polymer. *Angew. Chem. Int. Ed.* **55**, 13784–13788 (2016).
9. Ye, Z. *et al.* A new isomeric porous coordination framework showing single-crystal to single-crystal structural transformation and preferential adsorption of 1,3-

butadiene from C4 hydrocarbons. *Cryst. Growth. Des.* **17**, 2166–2171 (2017).

10. Liao, P., Huang, N., Zhang, W., Zhang, J. & Chen, X. Controlling guest conformation for efficient purification of butadiene. *Science* **356**, 1193–1196 (2017).
11. Du, S. *et al.* Ultramicroporous carbons featuring sub-Ångstrom tunable apertures for the selective separation of light hydrocarbon. *AIChE J.* **67**, e17285 (2021).
12. Zhang, Z., Tan, B., Wang, P., Cui, X. & Xing, H. Highly efficient separation of linear and branched C4 isomers with a tailor-made metal–organic framework. *AIChE J.* **66**, e16236 (2020).
